# Patient-derived three-dimensional cortical neurospheres to model Parkinson’s disease

**DOI:** 10.1371/journal.pone.0277532

**Published:** 2022-12-01

**Authors:** Waseem K. Raja, Esther Neves, Christopher Burke, Xin Jiang, Ping Xu, Kenneth J. Rhodes, Vikram Khurana, Robert H. Scannevin, Chee Yeun Chung

**Affiliations:** 1 Yumanity Therapeutics, Boston, MA, United States of America; 2 Department of Neurology, Brigham and Women’s Hospital and Harvard Medical School, Ann Romney Center for Neurologic Disease, Boston, MA, United States of America; 3 Harvard Stem Cell Institute, Cambridge, MA, United States of America; 4 Broad Institute of MIT and Harvard, Cambridge, MA, United States of America; University of Pécs Medical School, HUNGARY

## Abstract

There are currently no preventive or disease-modifying therapies for Parkinson’s Disease (PD). Failures in clinical trials necessitate a re-evaluation of existing pre-clinical models in order to adopt systems that better recapitulate underlying disease mechanisms and better predict clinical outcomes. In recent years, models utilizing patient-derived induced pluripotent stem cells (iPSC) have emerged as attractive models to recapitulate disease-relevant neuropathology *in vitro* without exogenous overexpression of disease-related pathologic proteins. Here, we utilized iPSC derived from patients with early-onset PD and dementia phenotypes that harbored either a point mutation (A53T) or multiplication at the α-synuclein/*SNCA* gene locus. We generated a three-dimensional (3D) cortical neurosphere culture model to better mimic the tissue microenvironment of the brain. We extensively characterized the differentiation process using quantitative PCR, Western immunoblotting and immunofluorescence staining. Differentiated and aged neurospheres revealed alterations in fatty acid profiles and elevated total and pathogenic phospho-α-synuclein levels in both A53T and the triplication lines compared to their isogenic control lines. Furthermore, treatment of the neurospheres with a small molecule inhibitor of stearoyl CoA desaturase (SCD) attenuated the protein accumulation and aberrant fatty acid profile phenotypes. Our findings suggest that the 3D cortical neurosphere model is a useful tool to interrogate targets for PD and amenable to test small molecule therapeutics.

## Introduction

More than 7 million Americans are suffering from neurodegenerative diseases, including Alzheimer’s Disease, Parkinson’s disease (PD), and Amyotrophic Lateral Sclerosis (ALS) [1]. There are no disease modifying therapies available for these disorders that can halt, slow or reverse disease progression. Predictive cellular models for human disease pathologies are critical for the development of effective therapeutics that may lead to successful clinical outcomes. Gene overexpression or knockdown models of neurological disease pathways in transformed or immortalized human cell lines have inherent limitations and often lead to unsuccessful outcomes in preclinical and clinical development [2]. Transgenic animal models of neurodegenerative disease offer only limited insight into human disease mechanisms and potential therapeutic approaches [3].

Induced pluripotent stem cells (iPSC) derived neuronal models provide a cellular model in which to study disease-relevant pathologies in patient neuronal cells. Integration of iPSC technology with genome editing to generate an isogenic control makes this approach unique and attractive, as disease-relevant mutations can be studied at endogenous expression levels in neuronal cells [4,5]. In addition, iPSC have unlimited renewal capabilities and can be differentiated into multiple neuronal-lineages, thereby providing a robust source of human neural cultures. Such a patient-derived cellular model may provide personalized drug-screening platforms that can be used to develop phenotypic assays and validate novel therapeutic targets in specific patients and test their efficacy [6]. Using patient-derived cells as a starting point also could allow for an *in vitro* personalized medicine approach, where the therapeutic effects of drugs can be tested at the individual patient cell level to identify and enrich for responders to particular treatments.

Combining iPSC technologies with three-dimensional (3D) cultures provides a multicellular spatial architecture to better approximate the native environment of the brain. Two basic approaches to creating 3D culture systems are a tissue engineering approach where the neural cells are cultured in biocompatible 3D scaffolds, and suspension cultures to culture aggregated stem cells and differentiate them as organoids or spheroids, also known as neurospheres [7,8]. Neurospheres can be generated from pre-differentiated neuronal stem cells (NSC) to reduce the duration of neuron maturation. The use of NSC as a starting point provides an advantage as the cells are pre-defined to neuronal fates and it is possible to generate a consistent culture with lower variability in cellular composition.

The accumulation of pathogenic forms of α-synuclein (α-syn) is one of the primary pathological hallmarks of PD and the phosphorylation of serine 129 of α-syn (pS129 α- syn) has been linked to PD and is enriched in Lewy bodies (LBs) [9]. A recent study suggests that LBs are crowded not only with aggregated proteins but with lipid membranes including vesicular structures and dysmorphic organelles [10]. The link between PD and the dysregulation of membrane lipids is growing stronger [11]. Studies have shown that dysregulation of monounsaturated fatty acids (MUFAs) can contribute to α-syn accumulation, while limiting MUFAs appears beneficial to α-syn-related pathology [12,13]. Stearoyl CoA Desaturase (SCD) introduces a double bond into 16- and 18-carbon fatty acyl-CoA molecules (C16:0, palmityl CoA and C18:0, stearoyl CoA) to produce MUFAs (C16:1n7, palmitoleic acid and C18:1n9, oleic acid) that are incorporated into diverse lipid species, such as phospholipids, triacylglycerides, or cholesterol esters [14]. Recent work from our group as well as others strongly suggests that targeting this pathway through SCD inhibition may prove to be therapeutic in alleviating several PD-related phenotypes seen in model systems [15–17].

Here, we described the development of a cortical neurosphere model from PD patient- derived iPSC either carrying the A53T mutation in the gene encoding α-syn, *SNCA* (α- syn A53T) or triplication of the *SNCA* locus (S3) paired with corresponding mutation- corrected isogenic control. Compared to their isogenic controls, neurospheres from the PD patients showed dysregulated fatty acid profiles and accumulated total and phosphorylated α-syn levels. These disease-relevant phenotypes were reversed by a stearoyl-CoA desaturase inhibitor, CAY10566.

## Results

### Generation and characterization of neural stem cells (NSC)

**Fig 1:**
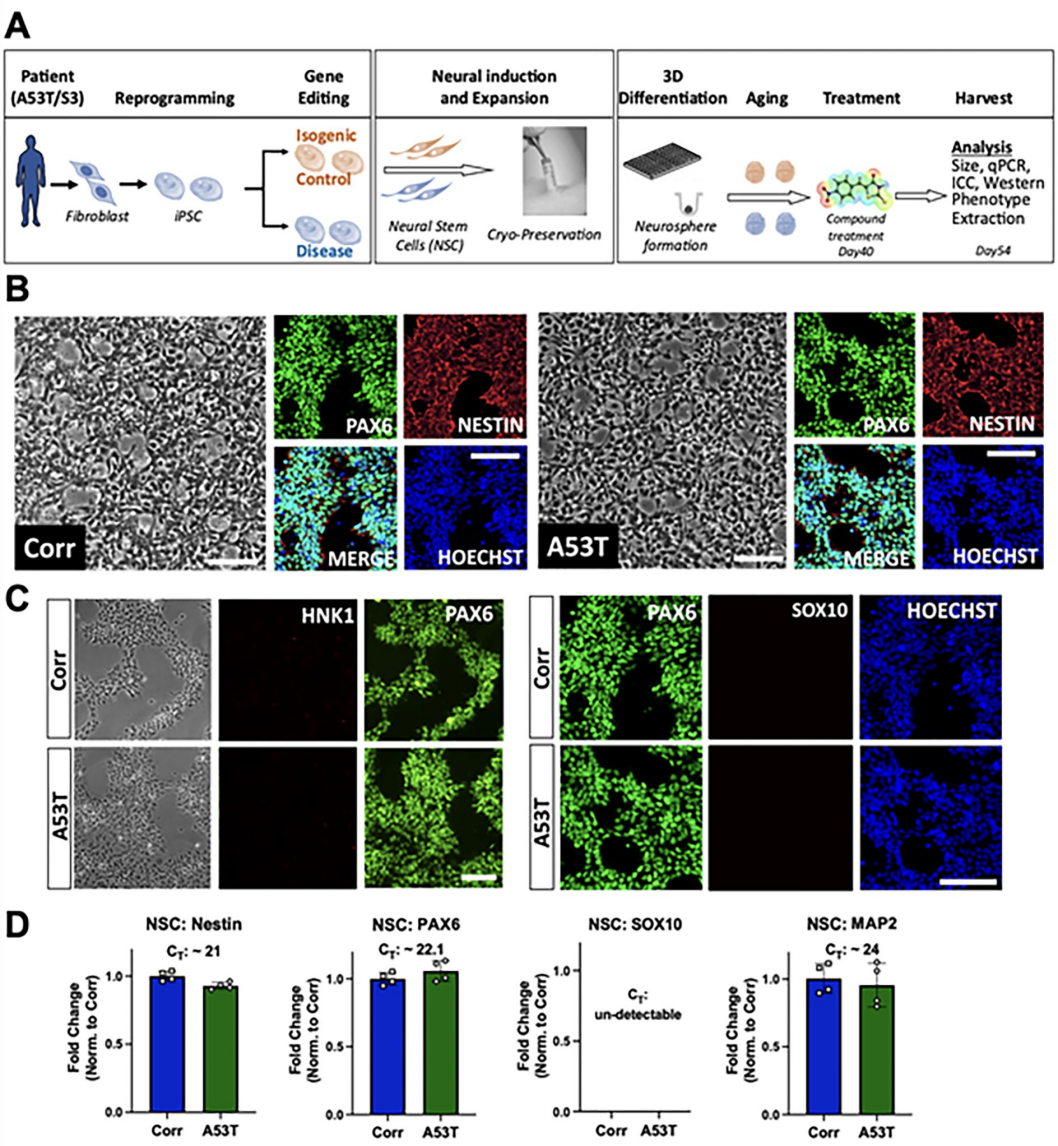
Process flow and quality control of Neural Stem Cells (NSC). **A**) Process flow of patient-derived neurosphere generation. **B**) Representative phase contrast images and immunocytochemistry (ICC) for Hoechst and NSC markers (Nestin and PAX6). Left Panel: Isogenic control line (Corr). Right panel: A53T line. Scale bar: 100 μm. **C**) ICC for Hoechst, PAX6, and neural crest cell markers (HNK1 and SOX10). Top panels: Isogenic control line (Corr). Bottom panels: A53T line. Scale bar: 100 μm **D**) Gene expression mRNA levels of Nestin, PAX6, SOX10 and MAP2 quantified using qPCR, normalized to a housekeeping control gene (GAPDH), and with fold changes measured relative to the isogenic control. qPCR data shows two biological replicates with two technical replicates each. Data is mean + standard deviation.

The iPSC were carefully maintained by manually removing spontaneously differentiated cells. The A53T iPSC line and an isogenic control line (Corr), in which the A53T mutation was corrected by CRISPR-Cas9 genome engineering, were differentiated into neural stem cells (NSC). After differentiation and purification (explained in the Materials and Methods section), initial quality control measures were implemented on both lines to assess the quality and purity of NSC. The NSC were banked and thawed for future experiments to generate two-dimensional or three-dimensional cultures. **Fig 1A** shows the entire process flow of the patient-derived *in vitro* model system utilized here. Phase contrast images of NSC showed similar morphology in both cell lines. Immunofluorescence microscopy also revealed similar staining patterns and levels of cell markers in both lines. Purified NSC were positive for neural stem cell markers such as PAX6 and Nestin (**Fig 1B**). Additionally, the neural crest cell markers HNK1 and SOX10 were undetectable at this stage (**Fig 1C)**. Quantitative RNA analysis confirmed the immunostaining results; there were elevated levels of forebrain progenitor marker PAX6 and neural progenitor marker Nestin in all NSC lines, along with undetectable levels of neural crest cell marker SOX10. We also detected the presence of MAP2 mRNA due to spontaneous differentiation of NSC into neurons (**Fig 1D)**.

The ability of neural stem cells to differentiate into neurons was then assessed as one of the quality controls measures. NSC (A53T and Corr) were plated on a poly-D-lysine/laminin coated surface and differentiated in neural differentiation media (referred to as “neurosphere differentiation media” in **S1 Table in S1 File**) in a 2D format. After differentiation and maturation, a clear neuronal morphology was observed with positive staining for the neuronal marker Tau and dendritic marker MAP2 in both A53T/Corr lines (**S1A Fig in S1 File**). Taken together, these results established that both the A53T and the isogenic control line (Corr) were capable of generating neural stem cells and neurons.

We also generated NSC from patient-derived iPSC harboring a triplication of the *SNCA* locus (S3) using the same protocol, along with the CRISPR-Cas9 generated isogenic control α-syn knockdown line (KD), where two copies of the *SNCA* genes were deleted to reduce α-syn levels closer to wild-type level. The NSC created from the S3 and KD iPSC were evaluated using the same quality control measures as described above. Phase contrast imaging showed similar morphology between the S3 and KD NSC (S2A Fig in **S1 File**). Immunofluorescence microscopy also shows positive staining for NSC markers PAX6 and Nestin, and negative staining for neural crest markers HNK1 and SOX10 in both S3 and KD lines. (**S2A and S2B Fig in S1 File)**. Quantitative RNA analysis also confirmed positive expression of PAX6 and Nestin, while no expression of SOX10 was observed (**S2C Fig in S1 File)**. Additionally, NSC derived from the S3 and KD lines successfully generated Tau and MAP2 positive neurons in a monolayer differentiation culture (**S1B Fig in S1 File)**.

### Generation and characterization of neurospheres

**Fig 2:**
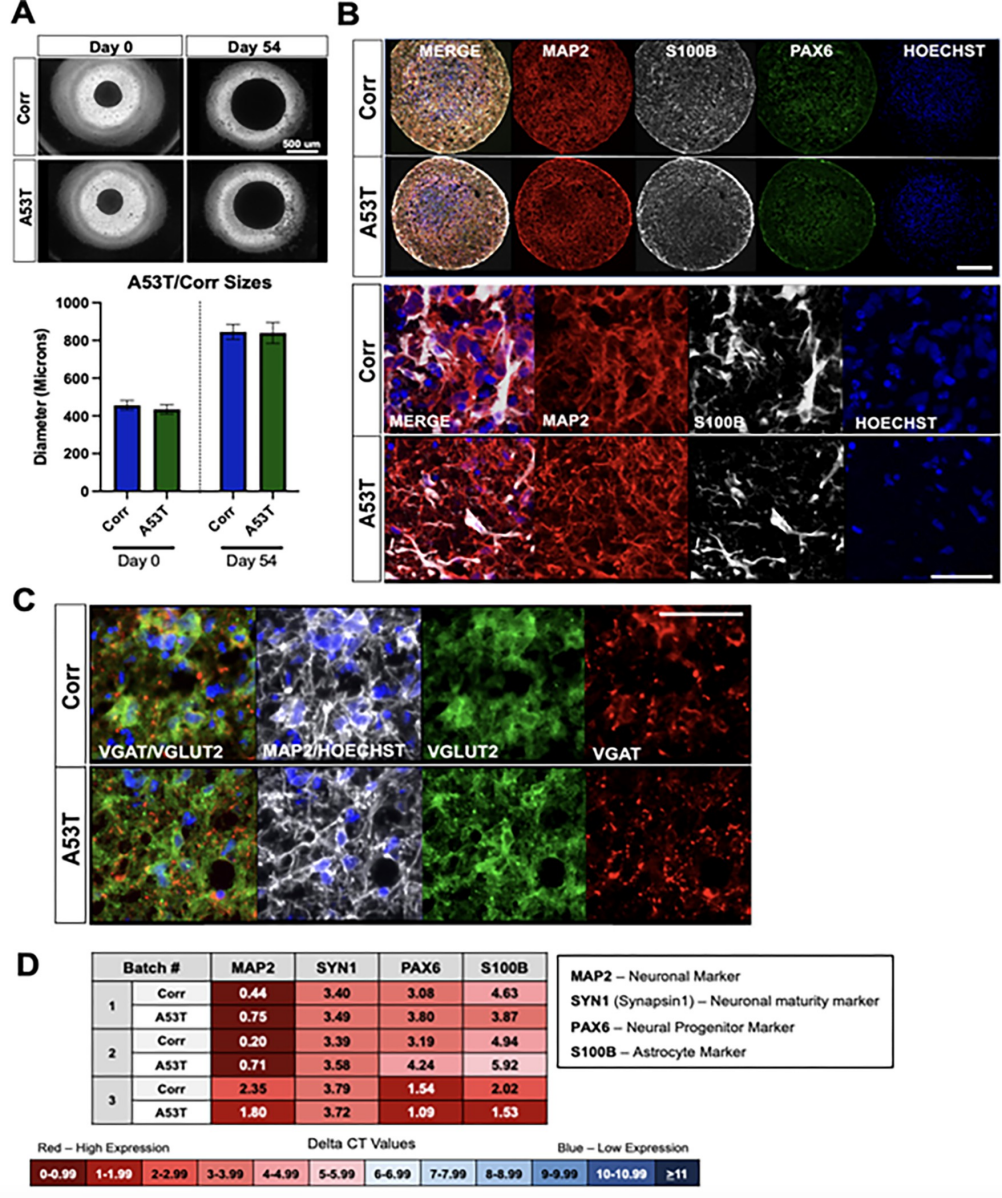
Quality control for A53T/Corr neurosphere differentiations shows cellular subtype composition of cultures. **A**) Size difference and variability of neurospheres. Top: Representative phase contrast images of A53T and Corr neurospheres at day 0 and day 54. Bottom: Quantified mean + standard deviation of individual neurospheres across three independent differentiations; n>20 for all groups. **B**) Immunocytochemistry (ICC) of 54-day-neurospheres for neuronal marker MAP2, astrocyte marker S100B, NSC marker PAX6, and Hoechst. Top panel: Whole sphere view of A53T and Corr spheres. Scale bar: 200 μm. Bottom Panel: Magnified view of MAP2 and S100B positive cells. Scale bar 50 μm. **C**) ICC of 54-day-neurospheres for MAP2, glutamatergic neuron marker VGLUT2, GABAergic neuron marker VGAT, and Hoechst. Scale bar: 50 μm. **D**) Gene expression of MAP2, PAX6, S100B, and Synapsin-I in 54-day-neurospheres for three representative neurosphere differentiations. Each differentiation is a pool of 80–90 neurospheres. Delta CT values, measured by qPCR, are the difference between the CT values of the housekeeping gene (GAPDH) and the gene of interest. A lower CT value (in red) indicates high gene expression, while a high CT value (in blue) indicates low gene expression.

Three-dimensional (3D) neurosphere cultures may provide a more relevant and complex environment to better mimic brain tissue. A protocol for generating neurospheres was therefore optimized for the A53T/Corr and the S3/KD neural stem cells. The neurospheres were formed in an ultra-low adhesion 384 well format. It was noted during the differentiation and maturation process that sphere size gradually increased based on quantitation of phase contrast images. Additionally, the well-to-well variability of the neurospheres sizes was low in each differentiation, making it easier to control the sizes between the disease and isogenic control lines (**Fig 2A** and **S3A Fig in S1 File**). qPCR analysis showed the appropriate mRNA expression of differentiation stage markers at iPSC, NSC and neuron stage (S4 Fig in **S1 File**). mRNA levels of relevant marker genes revealed no significant difference in levels of MAP2, Synapsin-I, S100B (astrocyte marker) and PAX6 in patient versus isogenic control neurospheres, while the MAP2 level was drastically increased in the 54-day-neurospheres compared to NSC (**Fig 2D, S3D and S4 Figs in S1 File)**. Immunofluorescence microscopy for MAP2 and S100B corroborated the RNA analyses for the A53T/Corr and S3/KD neurospheres (**Fig 2B and S3B Fig in S1 File**). The glutamatergic neuron markers VGLUT2, BRN2, mGLUR1, and TBR1 were abundantly expressed in these cultures as well as GABAergic neuron markers, VGAT, GABA and GAD1/67(**Fig 2C, S3C and S5 Figs in S1 File)**. These results suggest that the cortical neurospheres contain glutamatergic and GABAergic neurons, as well as astrocytes.

### Phenotypic assessment in NSC and neurospheres

#### Neurospheres from patient-derived iPSC display an aberrant fatty acid profile

Recent work from our group and others identified SCD as a potential target to ameliorate α-syn toxicity [15–17]. These studies implicate fatty acid biology in mediating aspects of α-syn toxicity, which prompted the evaluation of fatty acid profiles in neural stem cells and mature neurospheres derived from A53T/Corr and S3/KD lines.

**Fig 3:**
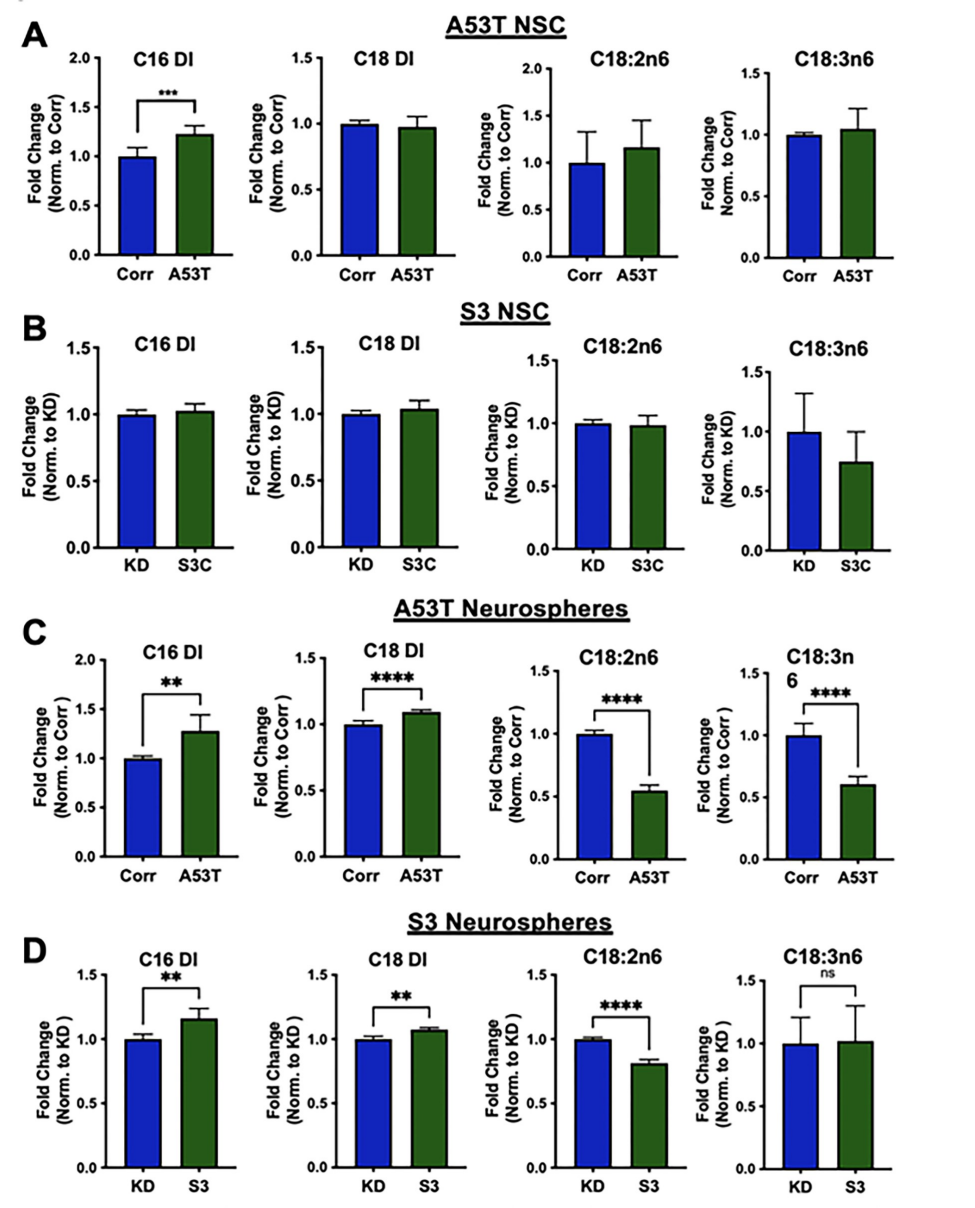
Fatty acid profile of NSC and neurospheres. Desaturation index (DI) for C16 (C16:1n7/C16:0) and C18 (C18:1n9/C18:0) along with the relative levels of the essential fatty acids, linoleic (C18:2n6) and γ-linolenic acid (C18:3n6), for the A53T/Corr NSC **(A**) and S3/KD NSC and **(B),** for the A53T/Corr neurospheres **(C**) S3/KD neurospheres **(D)** at day 54. The analysis was conducted using an unpaired two-tailed Student’s t-test, with n = 6 for **(A)** and n = 5 for **(B).** The analysis for the neurospheres was conducted using an unpaired t-test, with data from three different neurosphere differentiations. Each differentiation has two biological replicates (total 6 biological replicates). Data is mean + standard deviation and analyzed by a two-tailed Student’s t-test. (*P<0.05; **P<0.01; ***P<0.005; ****P<0.0001).

Twenty-six saturated and unsaturated fatty acids ranging from 14 to 22 carbon chain lengths were profiled in NSC from all lines, and little significant difference was observed between the disease lines (A53T and S3) and corresponding isogenic control lines (Corr and KD), with the exception of an elevated C16 desaturation index in the A53T NSC (**Fig 3A and 3B**). The C16 desaturation index was calculated as the ratio of palmitoleic acid (C16:1n7) to palmitic acid (C16:0). In neurospheres cultured for 54 days, there were several changes consistent between the disease (A53T and S3) lines and isogenic control lines. These included significant increases in both the C16 and C18 (C18:1n9/C18:0) desaturation indices in the A53T and S3 lines compared to the respective isogenic control lines **(Fig 3C and 3D)**. The level of an elongation product of C16:1n7, C18:1n7 was also significantly elevated in the disease lines compared to the control lines (**S6 and S7 Figs in S1 File**). In contrast, an essential fatty acid such as linoleic acid (C18:2n6) and polyunsaturated fatty acids (PUFA), such as γ-linolenic acid (C18:3n6) and docosapentaenoic acid (C22:5n3), were significantly reduced in the disease (A53T and S3) neurospheres as compared to the respective isogenic control (Corr and KD) neurospheres (**Fig 3C, 3D, S6 and S7 Figs in S1 File**). These data suggest that familial synucleinopathy mutations, including α-synuclein A53T mutation and triplication, influence fatty acid homeostasis in patient-derived neurons.

#### Patient-derived neurospheres display an increase in total and phospho-serine129 α-synuclein

**Fig 4:**
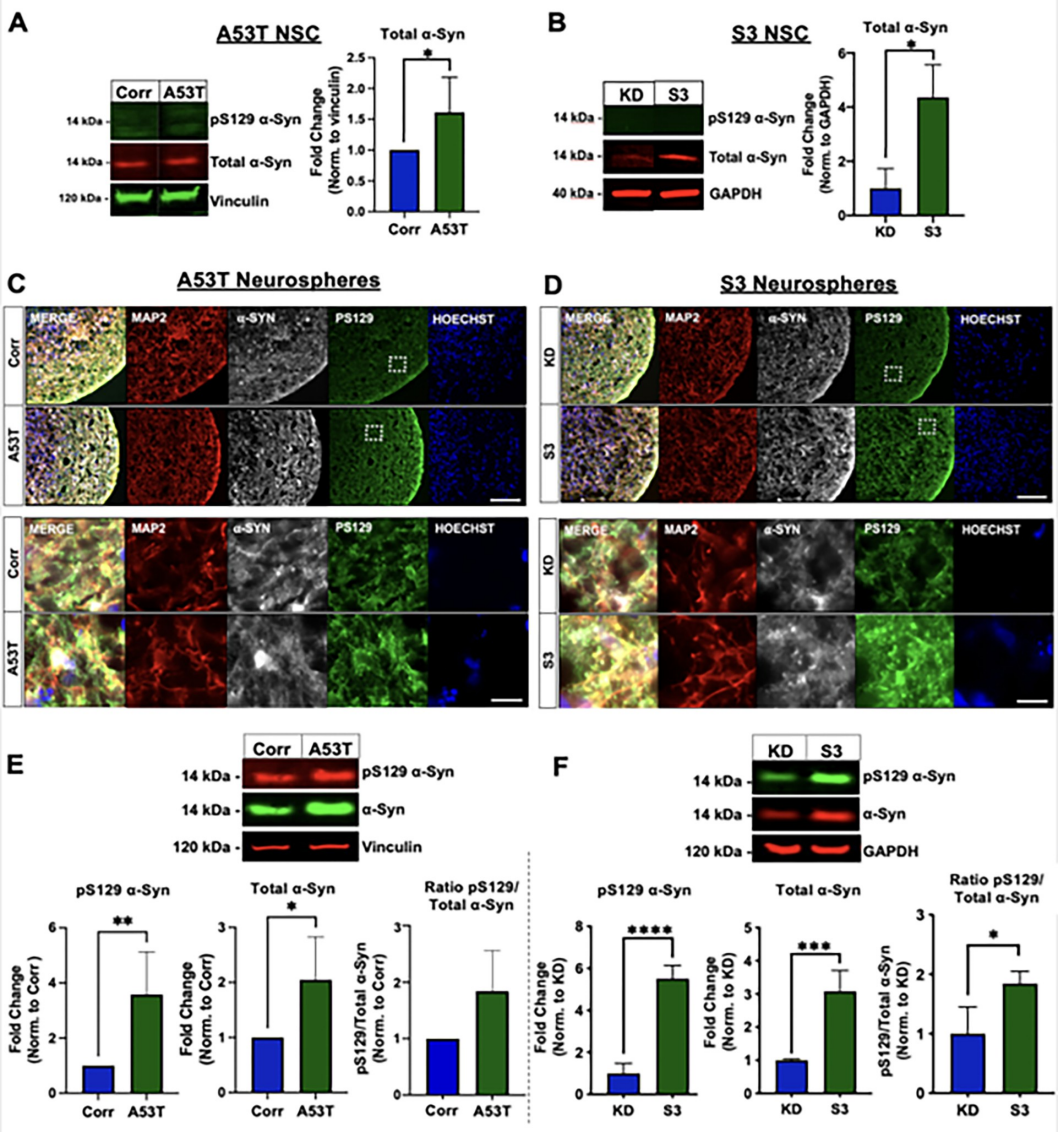
Total and pS129 α-synuclein in NSC and neurospheres. **A** and **B**) Representative Western blot for pS129 and total α-synuclein (left panels) and quantification (right panels) for total α-synuclein in the A53T/Corr NSC (**A**) and S3/KD NSC (**B**). **C** and **D**) Immunostaining of neurospheres at day 54 for MAP2, α-synuclein, pS129, and Hoechst. (**C**) A53T/Corr neurosphere view at low magnification (top, scale bar: 100 μm) and high magnification (bottom, scale bar: 25 μm). (**D**). S3/KD neurosphere view at low magnification (top, scale bar: 100 μm) and high magnification (bottom, scale bar: 25 μm). **E** and **F**) Representative Western blot (top panels) and quantification (bottom panels) for pS129, total α-synuclein, and pS129/total α-synuclein ratio for A53T/Corr (**E**) and S3/KD (**F**) neurospheres at day 54. For the NSC total α-synuclein quantification, n = 6 for A53T/Corr (**A**) and n = 3 for S3/KD (**B**). The neurosphere quantification data is an aggregate of five separate differentiations for the A53T/Corr lines (**E**), and three separate differentiations for the S3/KD lines (**F**). The S3/KD differentiations have two biological replicates within the differentiations (total 6 biological replicates), which result in error bars in the control groups, whereas the A53T/Corr groups do not. Data is mean + standard deviation and analyzed by a two-tailed Student’s t-test. (*P<0.05; **P<0.01; ***P<0.005; ****P<0.0001).

Increased levels of α-syn are linked to sporadic and familial PD as well as Lewy body dementia and multiple system atrophy [18]. In addition, phosphorylation of serine residue 129 (pS129) in α-syn has been linked to PD and is enriched in Lewy bodies [19], causing it to gain traction as a biomarker for the disease. Both α-syn and pS129 α-syn levels were measured in the NSC and the cortical neurospheres from these two pairs of PD patient lines using Western blot. At the NSC stage, pS129 α-syn was not detected in any of the lines. However, a significant increase in total α-syn levels was observed in the A53T and S3 lines compared to the Corr and KD lines **(Fig 4A and 4B).** After undergoing cortical neuron differentiation and 54-days of maturation, the neurosphere cultures displayed increased levels of total α-syn protein and a more dramatic increase in pS129 α-syn in the A53T neurospheres compared to the Corr control (**Fig 4C and 4E**). This phenotype was consistent throughout multiple rounds of differentiation. Additionally, we observed a similar phenotype in the S3 triplication line where the S3 neurospheres displayed an increase in both total α-syn and pS129 α-syn compared to the KD control neurospheres. Taking the ratio of pS129 α-syn to total α-syn, both the A53T and S3 lines show an increase in the α-syn ratio compared to their isogenic controls (**Fig 4D and 4F**).

### Effects of SCD inhibitor CAY10566 on A53T/Corr and S3/KD neurospheres

**Fig 5:**
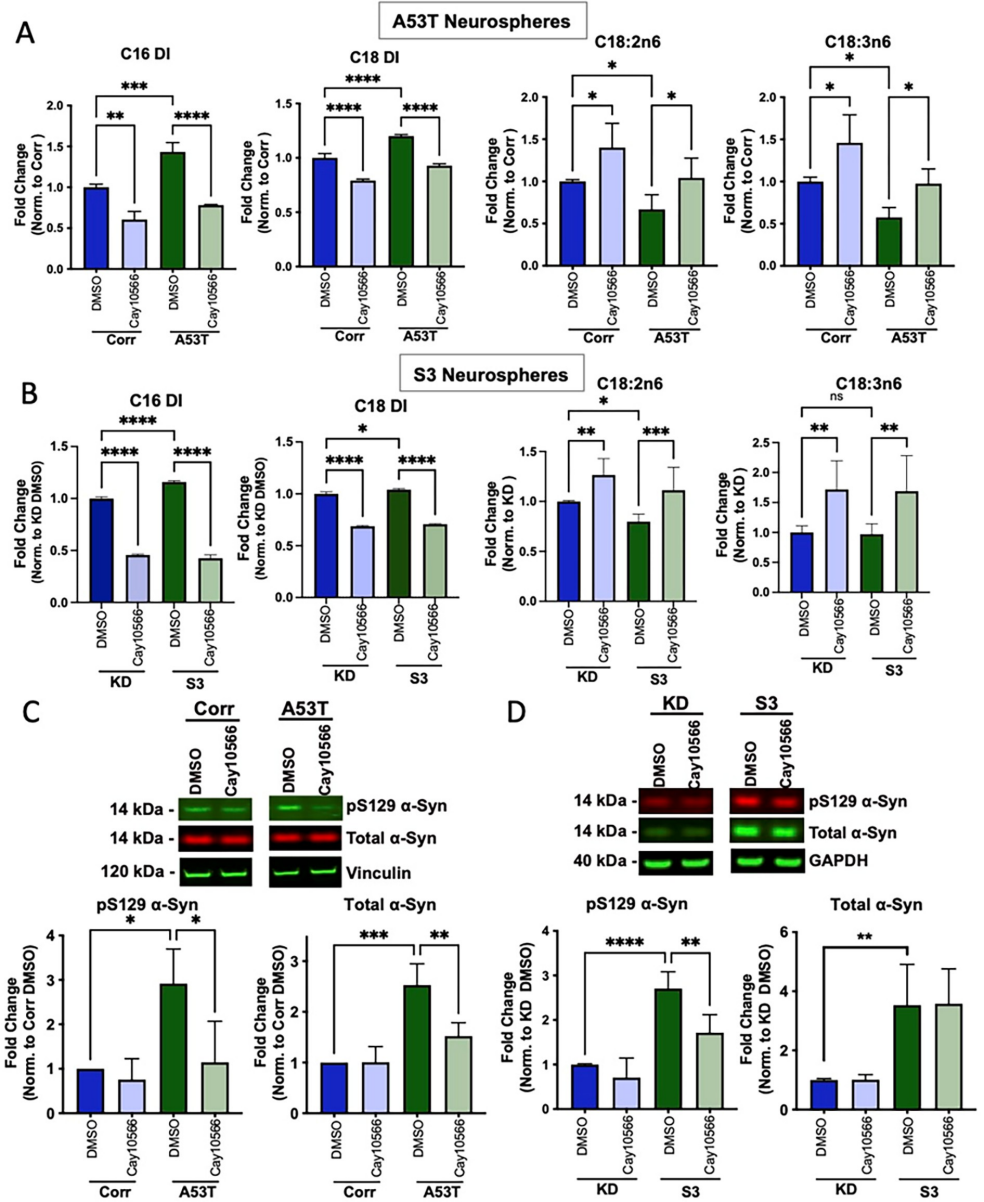
Treatment with the SCD inhibitor CAY10566 reversed the abnormal fatty acid profiles and pathogenic α-synuclein levels. **A** and **B**) C16 and C18 desaturation index (DI) and essential fatty acids Linoleic (C18:2n6) and γ-Linolenic acid (C18:3n6) from A53T/Corr neurospheres (**A**) and S3/KD neurospheres (**B**) treated for 2 weeks with 0.03% DMSO and 0.3 μM CAY10566. Data are from three independent neurosphere differentiations, with two biological replicates per differentiation, total 6 biological replicates. All values in each differentiation were normalized to the isogenic control groups treated with DMSO in each respective batch of differentiation. **C and D)** Representative Western blot (top panels) and quantification (bottom panels) for pS129 and total α-synuclein in 54-day-old cortical neurospheres from A53T/Corr (**C**) and S3/KD (**D**) treated for 2 weeks with 0.03% DMSO and 0.3μM CAY10566. Quantification includes data from three (A53T/Corr) and two (S3/KD) distinct differentiations with two biological replicates per group in the S3/KD neurosphere differentiations (total 4 biological replicates). Data is mean ± standard deviation and analyzed by One-way ANOVA analysis with Tukey’s multiple comparison test. (*P<0.05; **P<0.01; ***P<0.005; ****P<0.0001).

We then treated 40-day-old neurospheres with a potent SCD inhibitor CAY10566 to see if SCD inhibition can correct the change in fatty acid profiles in the disease lines. Treatment of A53T and S3 neurospheres with 0.3 μM CAY10566 for 14 days reversed the elevation of C16 and C18 DI in both lines (**Fig 5A and 5B**). Interestingly, the reduced levels of linoleic (C18:2n6) and γ-linolenic acids (C18:3n6) in the A53T line and linoleic acid (C18:2n6) in the S3 line were also reversed by CAY10566 (**Fig 5A and 5B**). A similar effect of CAY10566 was also observed in the isogenic control lines (**Fig 5A and 5B**). These results suggest that SCD inhibition alleviated the abnormal fatty acid profiles in PD patient-derived neurospheres.

The same treatment paradigm with CAY10566 also reduced total and pS129 α-syn levels in A53T neurospheres (**Fig 5C**). CAY10566 also resulted in a similar reduction of pS129 α-syn in the S3 lines (**Fig 5D**), while total α-syn levels were not greatly impacted in the S3/KD line. These results suggest that SCD inhibition attenuated the abnormal accumulation of α-syn in PD patient-derived neurospheres. Additionally, an analysis of Synapsin-I and ubiquitin protein levels in DMSO and CAY10566 treated S3/KD spheres revealed no significant changes in Synapsin-I and ubiquitin levels between the two groups in both S3 and KD lines (S8 Fig in S1 File). A decrease in Synapsin-I and increase in ubiquitin are indicators of degenerating neurospheres, and a lack of such in CAY10566 treatment indicates that the compound treatment was not toxic to the neurospheres.

## Discussion

Although the movement disorder later to be known as Parkinson’s disease (PD) was first described over 200 years ago, there is currently no effective therapy to stop, slow, or reverse the progression of the disease. The first step to tackle this problem is to develop predictive models that help us to recapitulate and understand the pathophysiology of the disease. This recapitulation of PD pathophysiology can then be harnessed to test the effects of therapeutic compound treatment.

As such, each model has its own inherent advantages and drawbacks. While post-mortem brain tissue from PD patients is an invaluable resource to identify both the hallmark pathologies of the disease, such as the identification of Lewy bodies, it offers only a viewpoint of the end stage of the disease and does not provide a model system that is tractable for biological and drug screening efforts. Engineered rodent models based on disease-linked mutations have helped understand the progression of pathology in animals, but the intrinsic differences between animals and humans may contribute to the lack of translation in efficacy from animals to the clinic [3]. Considering these limitations, using patient-derived cellular models generated by induced pluripotent stem cells (iPSC) may provide an attractive alternative approach [4,6]. Supporting this, many studies identified disease-relevant phenotypes from *SNCA* mutations (A53T point mutation, SNCA locus triplication) in iPSC derived neurons, such as increases in aggregated/ insoluble α-synuclein, oxidized dopamine, nitrosative stress, dysfunction in the lysosomal-autophagy pathway, vesicle trafficking, and mitochondrial abnormalities among others [20–27].

Patient-derived iPSC can be instructed towards an ectoderm lineage and differentiated into neural stem cells (NSC). Dual-SMAD inhibition (BMP and TGF-β signaling inhibition) is the most common technique used to generate NSC from iPSC, and a number of published literature has shown the utility and efficiency of this technique in both 2D (monolayer) and 3D (Embryoid body) differentiations. These discoveries led to the development of commercial kits to generate NSC from iPSC, one of which we used (Thermo Fisher) in our study to differentiate iPSC into NSC in a monolayer format. The NSC were further differentiated into neurons and astrocytes in a 3D suspension culture format without the use of any supporting biomaterial. One of the biggest advantages of the 3D neurospheres generated from these NSC is its batch to batch reproducibility, since the same batch of well characterized NSC are used for every differentiation. Additionally, the ease of compound treatment and scalability of the 3D cultures allow more cellular material to undergo high throughput screening. 3D cultures can also be aged more easily than 2D cultures, which tend to lift off from the coated cell culture surface during aging.

Although neurospheres provide a more accessible platform to age our neurons, they need careful monitoring and considerable quality control measures to determine the differentiation quality. Since the starting cells for the differentiation are NSC, the resulting cells are limited to a neural or astrocytic lineage. A typical differentiation yields a mix of glutamatergic (MAP2-, VGLUT2-, BRN1-, TBR1-, mGLUR1-positive) and GABAergic neurons (MAP2-, GAD1/67-, VGAT-, GABA-positive) with the presence of S100B-positive astrocytes. If the disease and control lines exhibit substantially different cellular subtype compositions, it can affect the downstream analyses and compound treatments. In that aspect, it is crucial to monitor the composition of cellular subtypes to ascertain that any phenotypes observed are due to genotypic differences, not differentiation differences between the lines. Overall, the neurosphere differentiation method yields cell cultures with consistent quality and robust disease-relevant phenotypes.

While still an emerging field, the link between Parkinson’s Disease and disrupted lipid biology is becoming stronger [11]. Our results add to this growing evidence by identifying the elevation of C16 and C18 DI along with other altered fatty acids in the A53T and S3 patient-derived neurospheres. The elevated levels of monounsaturated fatty acid (MUFA) could alter lipid homeostasis in the cells since these MUFAs are incorporated into various lipid species such as diacyl glyceride, triacyl glyceride, phospholipid, affecting membranes curvature, content of lipid droplet and even cell signaling [11]. This phenotype appears to be cell-type dependent, as no significant changes are observed at the NSC stage. Moreover, this aberrant lipid desaturation phenotype appears in both our A53T mutant and SNCA triplication lines, suggesting that dysregulation of lipids and altered fatty acid saturation may be a key pathological event occurring in synucleinopathies and common across different genetic forms of the disease.

The accumulation of pathogenic forms of α-syn is one of the primary pathological hallmarks of PD and the phosphorylation of serine 129 of α-syn has been linked to PD and is enriched in LBs [9]. These findings have been recapitulated in several models of PD, ranging from human α-syn overexpression models to iPSC neurons derived from PD patients harboring mutations in α-syn or PD risk factors [25,28–30]. Here, we have shown the accumulation of total and pS129 α-syn in PD patient-derived cortical neurospheres, consistent with previous studies across PD models. Interestingly, we observe that two-week treatment of SCD inhibition with CAY10566 not only reversed the aberrant fatty acid profiles but also reduced the accumulation of pS129 α-syn in aged neurospheres from both the A53T and the S3 patients. The consistent findings in two patient lines raise an intriguing possibility that SCD inhibition may alleviate these a-syn-related phenotypes in PD patients as seen in model systems [15–17].

Taken together, our cortical neurosphere protocol provides a useful and robust tool to develop therapeutics against devastating neurodegenerative diseases by enabling identification of disease-relevant phenotypes and testing therapeutic agents in patient-derived cells.

## Materials and methods

### Ethics statement on the patient fibroblast collected for the study

A53T fibroblasts were collected under BU protocol (#H-27479) and then cultured/reprogrammed/differentiated under MGH protocol (#2009P000775) and MIT COUHES protocol (#0807002834). The line was licensed from Whitehead to Yumanity. S3 lines were obtained from Coriell/Rutgers University repository under the agreement. This information has been added to the supplementary method section in **S1 File**.

### iPSC generation, editing and maintenance

All cells were maintained in the incubator at 37°C with 5% CO2. The A53T patient-derived iPSC were generated from fibroblasts obtained from a patient skin biopsy at Boston University. The fibroblast to iPSC reprogramming was performed at the Harvard Stem Cell Institute iPS Core following a microRNA-enhanced mRNA reprogramming method by combining Stemgent mRNA Reprogramming kit (00–0071) and Stemgent microRNA Booster Kit (00–0073) based on the manufacturer’s protocol. The α-Syn triplication patient cell line (S3) was purchased from the Coriell Institute for Medical Research. The iPSC were expanded and maintained in mTeSR1 medium from Stem Cell Technologies (see iPSC Media in S1 Table in **S1 File**). For more detailed method please refer to the supplementary Methods in **S1 File**.

### Generation of Neural Stem Cells (NSC)

The iPSC were differentiated into Neural Stem Cells using a monolayer method. Briefly, the iPSC were plated and kept in NSC induction media (called NSC Generation see **S1 Table in S1 File**) for seven days. On day seven, the cells were re-plated in NSC induction media, and switched to NSC expansion media (NEM, see **S1 Table in S1 File**) the next day. Neural crest cells were removed by Accutase (StemCell Technologies, Cat. No. 07920) treatment and passaging (see supplementary Methods in **S1 File** for details). After achieving a pure population of NSC, the NSC were expanded and frozen in Synth-a-freeze Cryopreservation Medium (Thermo Fisher, Cat. No. A1254201) (**Fig 1A** complete process flow).

### Differentiation of NSC into neurons in 2D

The NSC were maintained and expanded in Neural Expansion Medium. For 2D neuron differentiation, the NSC were plated in Neural Expansion Medium. The next day, neuron differentiation media was added to the NSC (the same as neurosphere differentiation media in **S1 Table in S1 File**). The cells were kept in this media for 10 to 12 days. On day 10–12, the neurons were terminally plated in the desired plate format. The culture was kept for 2 weeks in neuron maturation media (the same as neurosphere maturation media in **S1 Table in S1 File**). At the end of the experiment, the neurons were washed once with 1X DPBS (Thermo Fisher, Cat. No. 14190–144) and fixed in 4% paraformaldehyde (Fisher Scientific, Cat. No. 50-980-487) for immunocytochemistry.

### Generation of neurospheres

The NSC were passaged and plated as a single cell suspension in 384 well ultra-low adhesion spheroid plates (Corning, Cat. No. 3830) at a density of 20,000 cells per well. After loading cells, the plates were centrifuged at 300g for 3 minutes to aggregate the cells and to remove air bubbles. The next day, the Neural Expansion Medium was replaced with the neurosphere differentiation media (see **S1 Table in S1 File**). The differentiation media was changed every three days. After ten days of differentiation, the neurosphere differentiation media was replaced by the neurosphere maturation media (see **S1 Table in S1 File**). The media was changed bi-weekly for the next three weeks, and then once a week after day 30. At fixed harvesting times, the neurospheres were washed with 1X DPBS and stored in -80°C (for RNA, Western Blot, or FADI analysis) or fixed in 4% paraformaldehyde for cryosectioning (**Fig 1A**).

### Compound treatment

40 day-old neurospheres from both patient-derived and isogenic control lines were treated with 0.3 uM CAY10566 (Cayman Chemicals, Cat. No. 10012562) along with DMSO as a vehicle. The final concentration of DMSO in the media was 0.03%. The spheres were treated twice a week and harvested after two weeks of treatment. The spheres were harvested and pooled from multiple wells of the 384 well plates for different assays.

### RT-qPCR

NSC and neurospheres were processed for RNA extraction using the RNeasy Plus Mini Kit (Qiagen, Cat. No. 74134). The extracted RNA was converted to cDNA with the qScript cDNA Supermix (Quanta Biosciences, Cat. No. 95048–025) and the Mastercycler ep (Eppendorf) using RT-PCR. The cDNA was loaded in a 96 well or 384 well qPCR plate at a starting concentration of 20 ng/ul for all the samples. Gene expression was then analyzed with qPCR using the Taqman Fast Advanced Master Mix (Thermo Fisher, Cat. No. 44-449-64), Taqman Gene Expression Probes (Thermo Fisher, Cat. No. 4331182, 4448490), and the Step One Plus Real-Time PCR System (Applied Biosystems). The list of genes run for qPCR are in **S2 Table in S1 File**.

### Immunocytochemistry

NSC and neurons were cultured in poly-L-ornithine/laminin 96 well plates (Corning, Cat. No. 354657) and fixed in 4% paraformaldehyde (PFA). The NSC were stained for different neuronal stem cell markers (PAX6 (Invitrogen, Cat. No. 426600, 1:200 dilution) and Nestin (Abcam, Cat. No. ab22035, 1:500 dilution)), as well as the neural crest markers SOX10 (R&D Systems, Cat. No. AF2864, 1:20 dilution) and HNK1 (Sigma-Aldrich, Cat. No. C6680, 1:200 dilution). Neurons were stained for different neuronal markers (MAP2 (BioLegend, Cat. No. 822501, 1:1000 dilution), total Tau (Dako, Cat. No. A0024, 1:500 dilution), VGLUT2 (Synaptic Systems, Cat. No. 135403, 1:500 dilution), VGAT (Synaptic Systems, 131011, 1:300 dilution), BRN2 (Millipore Sigma, Cat. No. MABD51, 1:50 dilution), TBR1 (Millipore Sigma, Cat. No. AB2261, 1:100 dilution), mGLUR1 (Abcam, Cat. No. ab109450, 1:100 dilution), and GAD1 (Millipore Sigma, Cat. No. MAB5406, 1:500 dilution)) and an astrocyte marker (S100B (Sigma-Aldrich, Cat. No. S2532, 1:500 dilution)). The neurospheres were harvested as mentioned above and fixed in 4% PFA overnight at 4°C. The neurospheres were cryosectioned (by Histoserv Inc., MD) and stained using a published protocol [8]. The stained slides were imaged on a Nikon confocal microscope. The catalog number, sources, and dilutions of all the primary and secondary antibodies are listed in **S3 Table in S1 File.**

### Western blotting

The harvested neurospheres stored at -80°C were thawed and lysed according to a published protocol with modifications [23] and protein concentrations were measured using the Pierce BCA Protein Assay Kit (Thermo Fisher, Cat. No. 23227). The lysates were run on 4–12% SDS PAGE gels (Invitrogen, Cat. No. WG1403BOX), and dry transferred to 0.2 um PVDF membranes (Invitrogen, Cat. No. IB24001). The blots were stained with primary antibodies and Li-Cor secondary antibodies and were imaged on a Li-Cor Odyssey CLX. The catalog number and sources of all the primary and secondary antibodies are listed in **S3 Table in S1 File.**

### Fatty Acid Desaturation Index (FADI) analysis

The neurospheres were harvested and stored in 80% methanol at -80°C. The samples were shipped (on dry ice) to OmegaQuant LLC and processed for different fatty acids using gas chromatography (GC) with flame ionization detection. Total lipids were extracted from the NSC and neurospheres and hydrolyzed. Individual fatty acids were derivatized as methyl esters, and then assessed by gas chromatography with flame ionization detection. Fatty acid composition was expressed as a percent of total identified fatty acids. The abundance of different fatty acids was quantified using Microsoft Excel and plotted using GraphPad Prism software.

### Statistical analysis

The results are presented as the mean ± standard deviation. All statistical analyses were performed using GraphPad Prism 7.0 software. The p values were calculated with a two-tailed Student’s t-test and ONE-way ANOVA (Tukey’s test *P<0.05; **P<0.01; ***P<0.005; ****P<0.0001). Results shown here are the representative data from several experiments performed independently at different time points by multiple individuals.

## Supporting information

S1 File (RTF)

S1 Raw images (PDF)
