# Supplementary material for: Patient-derived three-dimensional cortical neurospheres to model Parkinson’s disease
Source: PLoS One. 2022 Dec 1;17(12):e0277532. doi: 10.1371/journal.pone.0277532 (PMC9714816; doi:10.1371/journal.pone.0277532)
Supplement: S1 File — (RTF) [file pone.0277532.s001.rtf]

Supporting information
Title 
Patient-Derived Three-Dimensional Cortical Neurospheres to Model Parkinson's Disease 
Authors and Affiliation 
Waseem K. Raja1*#$, Esther Neves1#, Christopher Burke1, Xin Jiang1, Ping Xu1, Kenneth J. Rhodes1†, Vikram Khurana2,3,4, Robert H. Scannevin1‡, Chee Yeun Chung1* 
Supplementary Figures 1-8

Supplementary Tables 1-3

Supplementary Methods


Supplementary Figure 1. Representative Immunocytochemistry (ICC) of 2D neurons differentiated from disease and isogenic control lines. A and B) The NSC were expanded, differentiated into neurons, terminally plated, and fixed after two weeks in culture for the A53T/Corr lines (A) and the S3/KD lines (B). The top and bottom panels in (A) and (B) represent the isogenic control and disease neurons, respectively. The neurons were stained for Tau (whole neuron staining), MAP2 and Hoechst. Scale bar: 100 ìm. 


Supplementary Figure 2. iPSC to Neural Stem Cell differentiation from S3 and KD lines. A) Representative phase contrast images and immunocytochemistry (ICC) for Hoechst and NSC markers (Nestin and PAX6). Top Panel: isogenic control line (KD). Bottom panel: S3 line. Scale bar: 100 µm. B) ICC for Hoechst and neural crest cell markers (HNK1 and SOX10). Top panel: isogenic control line (KD). Bottom panel: S3 line. Scale bar: 100 µm C) Gene expression mRNA levels of PAX6, Nestin, and SOX10 quantified using qPCR, normalized to a housekeeping control gene (GAPDH), and with fold changes measured relative to the isogenic control. qPCR data shows two biological replicates with two technical replicates each. Data is mean + standard deviation.
Supplementary Figure 3. Quality control for S3/KD neurosphere differentiations shows cellular subtype composition of cultures. A) Size difference and variability of neurospheres. Top: Representative phase contrast images of S3 and KD neurospheres at day 0 and day 54. Bottom: Quantified mean + standard deviation of individual neurospheres across three independent differentiations; n>30 for all groups. B) Immunocytochemistry (ICC) of 54-day-neurospheres for neuronal marker MAP2, astrocyte marker S100B, NSC marker PAX6, and Hoechst. Top panel: Whole sphere view of S3 and KD spheres. Scale bar: 200 µm. Bottom Panel: Magnified view of MAP2 and S100B positive cells. Scale bar 50 µm. C) ICC of 54-day-neurospheres for MAP2, glutamatergic neuron marker VGLUT2, GABAergic neuron marker VGAT and Hoechst. Scale bar: 50 µm. D) Gene expression of MAP2, PAX6, S100B, and Synapsin-I in 54-day-neurospheres for three representative neurosphere differentiations. Each differentiation is a pool of 80-90 neurospheres. Delta CT values, measured by qPCR, are the difference between the CT values of the housekeeping gene (GAPDH) and the gene of interest. A lower CT value (in red) indicates high gene expression, while a high CT value (in blue) indicates low gene expression.

Supplementary Figure 4. MAP2, PAX6, and SOX10 gene expression levels in iPSC, Neural Stem Cells (NSC) and day 54 neurospheres (NS). Delta CT values, measured by qPCR, are the difference between the CT values of the housekeeping gene (GAPDH) and the gene of interest. A lower CT value (in red) indicates high gene expression, while a high CT value (in blue) indicates low gene expression. Left side table are the representative values from A53T and Corr while the right side table is for S3 and KSupplementary Figure 5. Additional neuronal subtype markers in 54 day neurospheres. A) Immunocytochemistry (ICC) of 54-day-neurospheres for neuronal marker MAP2, glutamatergic marker VGLUT2, GABAergic marker GAD1/67, and Hoechst. Top panel: Isogenic control (Corr). Bottom panel: A53T disease line. Scale bar: 100 µm. B) ICC of 54-day-neurospheres for MAP2, inhibitory neurotransmitter GABA, and Hoechst. Top panel: Isogenic control (Corr). Bottom panel: A53T disease line. Scale bar: 100 µm. C) ICC of 54-day-neurospheres for glutamatergic markers TBR1, BRN2, and mGLUR1 in S3 neurospheres. Scale bar: 100 µm.


 


Supplementary Figure 6. Fatty acid profiles for all the fatty acids detected in the A53T and the Corr neurospheres at day 54. Relative levels of individual fatty acids were compared between A53T and Corr neurospheres. Corr data was used to normalize A53T results.  Data is represented as mean ± standard deviation. At least 3 independent experiments were done. The analysis was conducted using an unpaired two-tailed Student's t-test. (*P<0.05; **P<0.01; ***P<0.005; ****P<0.0001; n=5 independent repeats)


Supplementary Figure 7. Fatty acid profiles for all the fatty acids detected in the S3 and the KD neurospheres at day 54. Relative levels of individual fatty acids were compared between S3 and KD control neurospheres. Corr data was used to normalize A53T results.  Data is represented as mean ± standard deviation. At least 3 independent experiments were done. The analysis was conducted using an unpaired two-tailed Student's t-test (*P<0.05; **P<0.01; ***P<0.005; ****P<0.0001; n=5 independent repeats)      


Supplementary Figure 8. Treatment with Cay10566 does not reduce Synapsin-I or increase Ubiquitin expression compared to DMSO control groups. Representative Western blot (top panel) and quantification (bottom panel) for Synapsin-I and Ubiquitin for 54-day S3/KD neurospheres. The S3 and KD neurospheres were treated with 0.03% DMSO and 0.3 µM Cay10566 for 2 weeks. Data is represented as mean + standard deviation; n=4 for Synapsin and n=2 for Ubiquitin. The analysis was conducted using a two tailed Student's t-test.


Supplementary Table 1. Media composition and information. All media and supplements used in the generation/maintenance of NSC and neurosphere are shown. 

Supplementary Table 2: List of TaqMan Probes used for quantitative real-time PCR Supplementary Table 3. List of primary and secondary antibodies used for Immunostaining and Western blotting. 

Supplementary Methods
iPSC generation, Editing and Maintenance 
All cells were maintained in the incubator at 37°C with 5% CO2. The A53T patient-derived iPSC were generated from fibroblasts obtained from a patient skin biopsy at Boston University. The fibroblast to iPSC reprogramming was performed at the Harvard stem cell core following a microRNA-enhanced mRNA reprogramming method by combining Stemgent mRNA Reprogramming kit (00-0071) and Stemgent microRNA Booster Kit (00-0073) based on the manufacturer's protocol.  The á-syn triplication patient cell line (S3) was purchased from the Coriell institute for medical research (iPSC: ND34391, patient fibroblast: ND27760). The iPSC were expanded and maintained in mTESR media from stem cell technology (see iPSC Media in Supplementary Table 1). 
The disease-causing A53T mutation was corrected in house using the CRISPR-Cas9 system to generate an isogenic control pair. Multiple clones were evaluated and confirmed to have a normal karyotype. Similarly, the S3 mutation was corrected in house using the same method to generate an isogenic control for the S3 iPSC. Briefly, guide RNAs were designed using the Zhang Lab Guide Design Tool (http://crispr.mit.edu) and cloned into pSpCas9(BB)-2A-GFP (PX458) (Addgene, Plasmid #48138). All clones were Sanger sequenced to verify inserted gRNA sequences. gRNA cleavage efficiencies were tested in 293T cells and analyzed by TIDE software (http://shinyapps.datacurators.nl/tide/) Guide 5' GTGGTGCATGGTGTGGCAAC 3' was used for A53T correction. A 127 nt ssODN with sequence 5' ATTTCATAGGAATCTTGAATACTGGGCCACACTAATCACTAGATACTTTAAATATCA TCTTTGGATATAAGCACAATGGAGCTTATCTGTTGCCACACCATGCACCACTCCCT CCTTGGTTTTGGAG 3' were synthesized at IDT and used as correction template. 2 ug pX458 plasmid and 1 ug ssODN was transfected to 1x 10^6^ A53T patient iPSCs using Lipofectamine 3000 (Thermo Fisher, Cat. No. L3000008) and sorted for GFP positivity. Sorted cells were plated on Matrigel (Corning, Cat. No. 354277) coated surface at 150 cell/cm2 to allow the formation of single-cell derived clones. After 7-10 days of culture, colonies were picked into 96 w/p, for expansion and genotyping. Genomic DNA of each clone was prepared using Zygem prepGEM Universal kit (VWR, Cat. No. 76218-666). Targeted region was PCR amplified using Forward primer 5' GCCCCGGTGTTATCTCATTCT and Reverse primer 5' TGTCCAAGGGTGTTTCCTGA. PCR products were Sanger sequenced using sequencing primer 5' GCAGTTTGTCAATACATTTTTGG. Clones with A53T correction along with a few non-targeted clones were expanded, banked and karyotyped. 
Similarly, the á-syn triplication S3 line was targeted with tested gRNA 5' GTAAAGGAATTCATTAGCCA. Targeted region was PCR amplified using Forward primer 5' TAGCCAAGATGGATGGGAGATG and Reverse primer 5' CCATCACTCATGAACAAGCACC. PCR products were Sanger sequenced using sequencing primer 5' AGAGTCTCACACTTTGGAGGGT. Sequencing results were analyzed by TIDE software (http://shinyapps.datacurators.nl/tide/). Clones with indels were further TOPO cloned to resolve the allelic editing event. Final clones with two copy SNCA deletion, resulting from frame-shift causing indels, were expanded, banked and karyotyped using G-banding (Cell Line Genetics, WI). 

Generation of Neural Stem Cells (NSC) 
On day zero of differentiation, the iPSC (both disease and corrected isogenic control) were lifted from the surface as a single cell suspension using Accutase (StemCell Technologies, Cat. No. 07920). After resuspending the iPSC, the Accutase was diluted using 1X DPBS (Thermo Fisher, Cat. No. 14190-144) and the suspension was centrifuged to obtain the cell pellet. This DPBS Accutase dilution process is referred to as “Accutase and DPBS” in this manuscript. The supernatant was aspirated, and the iPSC were re-plated onto a Matrigel (Corning, Cat. No. 354277) coated 6 well plate in NSC Generation Media (see Supplementary Table 1) with Rock Inhibitor (Peprotech, Cat. No. 1293823). On day one, the culture should be approximately 20% confluent. The media was replaced with NSC Generation Media without Rock inhibitor, followed by replenishing the media every other day until day seven. Any non-neural or unwanted colonies were manually scraped away. On day seven of differentiation, the cells were suspended into a single cell suspension using Accutase and 1X DPBS, and re-plated at a density of 2 x 10^5^ cells/cm2 on a Matrigel coated 6 well plate in NSC Generation Media with Rock inhibitor. The following day, the media was switched to Neural Expansion Media (see Supplementary Table 1). 
The NSC were examined under the microscope for unwanted neural crest cells (larger and flatter cells). The neural crest cells were removed by Accutase treatment. Briefly, the cells were washed with 1X DPBS, incubated in Accutase for two minutes at 37oC, then transferred to a microscope at room temperature. The cells were observed under the microscope and kept in the Accutase until all the flat cells have lifted off the plate. Once all the neural crest cells had lifted, the Accutase was aspirated, and the culture was gently rinsed with 1X DPBS three times. The remaining cells were suspended into a single cell suspension in NEM with Rock inhibitor and re-plated at 2 x10^5^ cells/cm2 on a Matrigel coated plate. The following day, the media was replaced with fresh NEM without Rock inhibitor, and the cultures were monitored for neural crest cells under the microscope for the next few days. If neural crest cells were observed, the Accutase cleaning step was performed as described above. After achieving a pure population of NSC, the NSC were transferred onto poly-L-ornithine/laminin (Corning, Cat. No. 354658) or poly-D-lysine/laminin (Corning, Cat. No. ​​354413; Sigma-Aldrich, Cat. No. L2020) coated surfaces. The NSC were expanded and frozen in Synth-a-freeze Cryopreservation Medium (Thermo Fisher, Cat. No. A1254201) (Figure 1A show the process flow). 
Differentiation of NSC into neurons in 2D 
The NSC were maintained and expanded in NEM. For 2D neuron differentiation, the NSC were plated on a poly-D-lysine/laminin (Corning, Cat. No. 356539; Sigma-Aldrich, Cat. No. L2020) coated surface in Neural Expansion Media. The next day, neuron differentiation media (the same as neurosphere differentiation media in Supplementary Table 1) was added to the NSC. The cells were kept in this media for ten to twelve days, with a media change every other day. The neurons were re-plated onto a new poly-L-ornithine/laminin 96 well plate (Corning, Cat. No. 354657​​). The neurons were aged for 2 to 3 weeks in neuron maturation media (the same as neurosphere maturation media in Supplementary Table 1). At the end of the experiment, the neurons were washed once with 1X DPBS and fixed in 4% paraformaldehyde for immunocytochemistry. 
Generation of neurospheres 
To plate the NSC for neurosphere differentiation, NSC were lifted from the surface as a single cell suspension using Accutase. After washing out Accutase, the NSC were resuspended in NEM and strained through a 40-micron cell strainer (Greiner Bio-one, Cat. No. 542040). The density of the cell suspension was adjusted to plate 20,000 cells per well. The cell suspension was added to the 384 well ultra-low adhesion spheroid plates (Corning, Cat. No. 3830), leaving the outermost rows and columns filled with sterile water. After loading cells, the spheroid plates were centrifuged at 300g for 3 minutes to aggregate the cells in the bottom of the well and to remove any air bubbles in the media. The NSC formed spheroids overnight in each well. The next day, around 80% of the NEM was aspirated and replaced with the neurosphere differentiation media (see Supplementary Table 1) to initiate differentiation. The differentiation media was changed every two or three days. After ten days of differentiation, the neurosphere differentiation media was replaced by the neurosphere maturation media (see Supplementary Table 1). The media was changed bi-weekly for the next three weeks, and then once a week after day 30. The total media volume was approximately 90 ìl per well in the 384 well plates. In each media change, 60 ìl was aspirated and the same volume was added back to each well. The neurospheres were harvested at different time points using wide orifice pipette tips (Thomas Scientific, Cat. No. 1148B68) for characterization. The desired number of spheres were added to microfuge tubes, washed with 1X DPBS and stored in -80°C (for RNA, protein, or FADI analysis) or fixed in 4% paraformaldehyde for cryosectioning (Figure 1A show the process flow). 
Compound treatment 
40 day-old neurospheres from both patient-derived and isogenic control lines were treated with 0.3 uM CAY10566 (Cayman Chemicals, Cat. No. 10012562) along with DMSO as a vehicle. The final concentration of DMSO in the media was 0.03%. The spheres were treated twice a week and harvested after two weeks of treatment. The spheres were harvested and pooled from multiple wells of the 384 well plates for different assays. The spheres were harvested and washed once with the 1X DPBS and processed for the appropriate assay. 
RT-qPCR 
NSC and neurospheres were washed once with 1X DPBS and processed for RNA extraction using the RNeasy Plus Mini Kit (Qiagen, Cat. No. 74134) and a hand-held tissue homogenizer (PRO Scientific, Bio-Gen PRO200 Homogenizer). The amount of RNA was quantified using Nanodrop (Thermo Fisher). The extracted RNA was converted to cDNA with the qScript cDNA Supermix (Quanta Biosciences, Cat. No. 95048-025) and the Mastercycler ep (Eppendorf) using RT-PCR. The cDNA was loaded in a 96 well or 384 well qPCR plate at a starting concentration of 20 ng/ul for all the samples. Gene expression was then analyzed with qPCR using the Taqman Fast Advanced Master Mix (Thermo Fisher, Cat. No. 44-449-64), Taqman Gene Expression Probes (Thermo Fisher, Cat. No. 4331182, 4448490), and the Step One Plus Real-Time PCR System (Applied Biosystems). The list of genes run for qPCR were PAX6, Nestin, SOX10, MAP2, S100B, and Synapsin-I, and were normalized with the housekeeping gene GAPDH. The catalog numbers of all the TaqMan probes are listed in Supplementary Table 2. 
Immunocytochemistry 
NSC and neurons were cultured in poly-L-ornithine/laminin 96 well plates (Corning, Cat. No. 354657​​), washed once with 1X DPBS, fixed for 30 minutes in 4% paraformaldehyde (PFA) at room temperature, and stored at 4°C in 1X DPBS with 0.05% sodium azide. The NSC were stained for different neuronal stem cell markers (PAX6 (Invitrogen, Cat. No. 426600, 1:200 dilution) and Nestin (Abcam, Cat. No. ab22035, 1:500 dilution)), as well as the neural crest markers SOX10 (R&D Systems, Cat. No. AF2864, 1:20 dilution) and HNK1 (Sigma-Aldrich, Cat. No. C6680, 1:200 dilution). Neurons were stained for different neuronal markers (MAP2 (BioLegend, Cat. No. 822501, 1:1000 dilution), total Tau (Dako, Cat. No. A0024, 1:500 dilution), VGLUT2 (Synaptic Systems, Cat. No. 135403, 1:500 dilution), VGAT (Synaptic Systems, 131011, 1:300 dilution), BRN2 (Millipore Sigma, Cat. No. MABD51, 1:50 dilution), TBR1 (Millipore Sigma, Cat. No. AB2261, 1:100 dilution), mGLUR1 (Abcam, Cat. No. ab109450, 1:100 dilution), and GAD1 (Millipore Sigma, Cat. No. MAB5406, 1:500 dilution)) and an astrocyte marker (S100B (Sigma-Aldrich, Cat. No. S2532, 1:500 dilution)). The neurospheres were harvested as mentioned above and fixed in 4% PFA overnight at 4°C. The next day, the PFA was washed out and neurospheres were stored at 4°C in 1X PBS with 0.05% sodium azide. The neurospheres were cryosectioned into 14 ìm sections on glass slides (by Histoserv Inc., MD). The sections were stained using a published protocol [1] (Raja et al., 2016). Briefly, the sections were washed once with 1X PBS, permeabilized for 30 minutes at room temperature in 1X PBS containing 0.3% Triton-X100 (Sigma-Aldrich, Cat. No. 93443-100ml) (PBST) and blocked for 1 hour at room temperature in PBST with 10% v/v normal Goat serum (Sigma-Aldrich, Cat. No. G9023). The blocking solution was replaced with the primary antibody solution (PBST with 5% v/v normal Goat serum and diluted primary antibody) and incubated overnight at 4°C. The next day, the sections received three 15 min washes in PBST containing 5% v/v normal Goat serum. After the last wash, the secondary antibody solution (PBST with 5% v/v normal Goat serum with diluted secondary antibody and Hoechst) was added to the sections. The sections were incubated in the secondary antibody solution for 2 hours at room temperature. The sections were washed three times in PBST containing 5% v/v normal Goat serum, as described before. Sections were then cover slipped in Fluoromount-G mounting medium (Thermo Fisher, Cat. No. 00-4958-02) and the edges were sealed with clear nail polish. Slides were imaged on a Nikon confocal microscope (Nikon, Eclipse Ti-E & C2/C2si). The catalog number and sources of all the primary and secondary antibodies are listed in Supplementary Table 3. 
Western blotting 
Approximately 60 to 80 neurospheres (or NSC pellets of 4-10 million cells) were harvested and stored at -80°C as mentioned above. The neurospheres were homogenized in lysis buffer (20 mM HEPES, 150 mM NaCl, 10% Glycerol, 1 mM EGTA, 1.5 mM MgCl2, 1% Triton X-100 (Sigma-Aldrich, Cat. No. 93443-100ml), protease inhibitor (Sigma-Aldrich, Cat. No. P8340) and phosphatase inhibitors (Sigma-Aldrich, Cat. No. P2850, P5726)) using a pestle homogenizer (Glas-Col, Stirrer Motor with Electronic Speed Controller 2224). After homogenization, the samples were incubated on ice for 20 minutes and subjected to two quick freeze-thaw cycles on a dry ice/ethanol slurry and a 37oC heat block Following the freeze-thaw, samples were centrifuged at 14,000g for 30 minutes at 4oC to sediment insoluble material. Supernatant protein was harvested and measured using the Pierce BCA Protein Assay Kit (Thermo Fisher, Cat. No. 23227). 40 ug of total protein was reduced using Laemmli SDS-Sample Buffer (Boston BioProducts, Cat. No. BP-110R), separated on 4-12% SDS PAGE gels (Invitrogen, Cat. No. WG1403BOX) in NuPAGE MES SDS Running Buffer (Thermo Fisher, Cat. No. NP0002), and dry transferred to 0.2 uM PVDF membranes (Invitrogen, Cat. No. IB24001). The membranes were fixed in 1% paraformaldehyde for 40 minutes at room temperature, rinsed three times with 1X TBS, and then blocked for 30 minutes at room temperature on a rocker (Li-Cor Intercept blocking buffer, Cat. No. 927-60001). The membranes were incubated in primary antibodies diluted in the Intercept buffer overnight at 4oC with gentle rocking. Following primary antibody incubation, blots were washed three times for 15 minutes each in a 1X TBS solution containing 0.01% Tween20 (Sigma-Aldrich, Cat. No. P1379). The membranes were then incubated in secondary antibodies diluted in the Intercept buffer at room temperature for 2 hours, followed by three washes in 1X TBS with 0.01% Tween20. After the washes, the blots were imaged on a Li-Cor Odyssey CLX and analyzed on Li-Cor ImageStudio software. The catalog number and sources of all the primary and secondary antibodies are listed in Supplementary Table 3. 
Data normalization and error bars: sometimes (e.g. A53T/Corr samples) but not all (e.g S3/KD samples), data points were generated by separate Western blot experiments of neurospheres from independent batches (biological replicates). Each Western blot run will produce variable raw signal levels that are mostly due to the run to run technical differences of immunoblotting (primary and secondary antibody strength and exposure time, etc). Variabilities caused by these factors are not of biological significance. To be able to fairly compare data from separate Western blots runs, the values of each blot were normalized to the housekeeping protein first (to account for protein loading differences) and then to the value of the isogenic control on the blot (to normalize against the raw signal difference introduced by immunoblotting procedure).  For example, A53T/Corr biological replicates were analyzed in independent Western blot runs and raw signal from A53T sample was normalized by a corr sample from the same Western blot run. Therefore, when pooling the data, all the Corr values average to “1” without any error bars. This was not the case with the S3/KD data, in which there were at least 2 biological replicates of isogenic controls and patient samples that were run on the same blot, resulting in error bars across all group.
Fatty Acid Desaturation Index (FADI) Analysis 
The neurospheres were harvested as mentioned above, washed once with 1X DPBS, resuspended in 350 ìl cold 80% methanol, and stored at -80°C until ready to process. The samples were shipped (on dry ice) to OmegaQuant LLC and processed for different fatty acids using gas chromatography (GC) with flame ionization detection. The neurosphere solution (80% methanol) was transferred into a screw-cap glass vial and dried in a speed vac. After drying, methanol containing 14% boron trifluoride was added and the vials were briefly vortexed and heated in a 100°C hot bath for 10 minutes. After cooling, hexane and HPLC grade water were added sequentially. The vials were recapped, vortexed, and centrifuged to separate layers. An aliquot of the hexane layer was transferred to a GC vial and processed. Fatty acid composition was expressed as a percent of total identified fatty acids. The abundance of different fatty acids was quantified using Microsoft Excel and plotted using GraphPad Prism software. 
Statistical Analysis 
The results are presented as the mean ± standard deviation. All statistical analyses were performed using GraphPad Prism 7.0 software. The p values were calculated with a two-tailed Student's t-test or One way ANOVA (Tukey's test *P < 0.05, **P < 0.01, ***P < 0.005; ****P < 0.0001). Results shown here are the representative data from several experiments performed independently at different time points by multiple individuals. 
Supplementary References 
1.	Raja, W.K., Mungenast, A.E., Lin, Y.T., Ko, T., Abdurrob, F., Seo, J., and Tsai, L.H. (2016). Self-Organizing 3D Human Neural Tissue Derived from Induced Pluripotent Stem Cells Recapitulate Alzheimer's Disease Phenotypes. PLoS One 11, e0161969.
Raja, W.K., Mungenast, A.E., Lin, Y.T., Ko, T., Abdurrob, F., Seo, J., and Tsai, L.H. (2016). Self-Organizing 3D Human Neural Tissue Derived from Induced Pluripotent Stem Cells Recapitulate Alzheimer's Disease Phenotypes. PLoS One 11, e0161969. 
