# Supplementary material for: Patient-derived three-dimensional cortical neurospheres to model Parkinson’s disease
Source: PLoS One. 2022 Dec 1;17(12):e0277532. doi: 10.1371/journal.pone.0277532 (PMC9714816; doi:10.1371/journal.pone.0277532)
Supplement: S1 Raw images — (PDF) [file pone.0277532.s002.pdf]

Figure 4A: pS129 α-Syn Panels
A53T – Disease NSC
Corr – Isogenic Control NSC
Imaged with LiCor Odyssey Dlx System

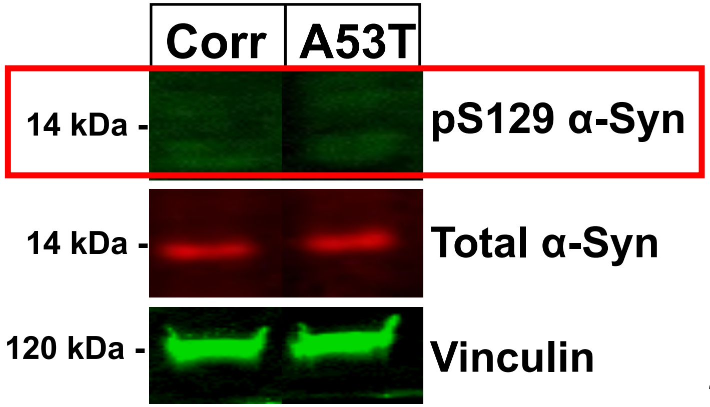

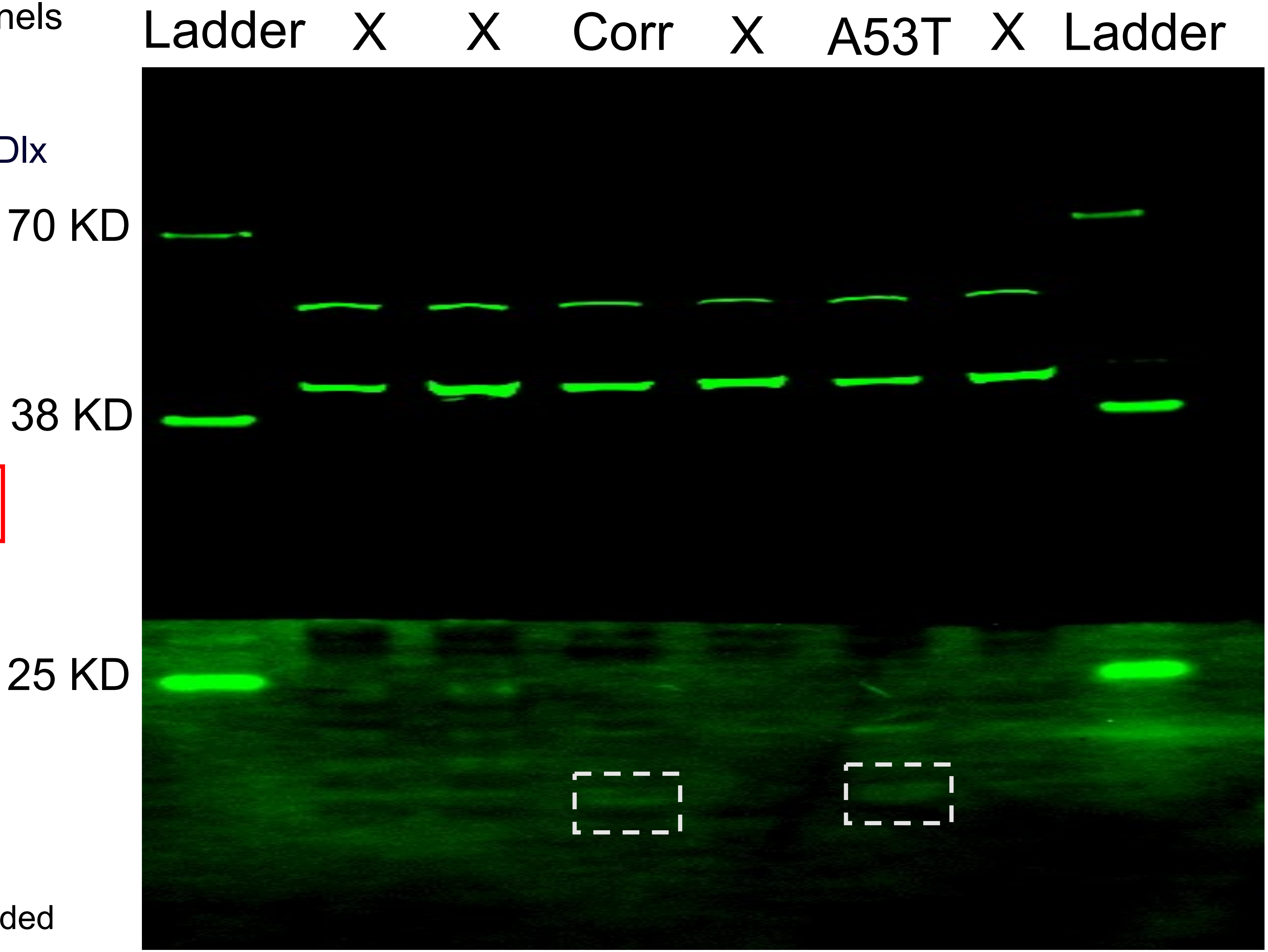

Blot was cut, both pieces included

Figure 4A: Total α-Syn Panels
A53T – Disease NSC
Corr – Isogenic Control NSC
Imaged with LiCor Odyssey DIx System

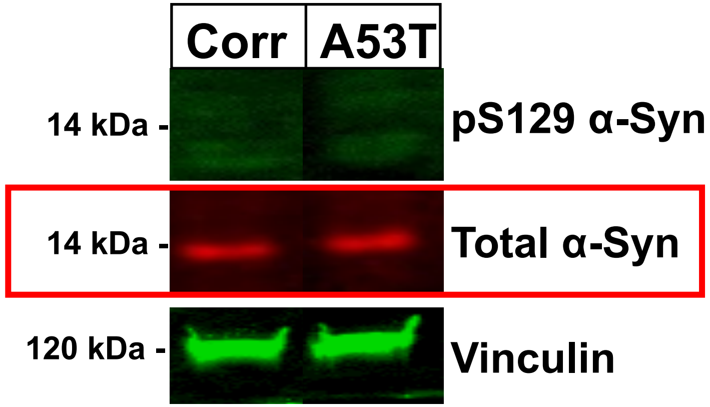

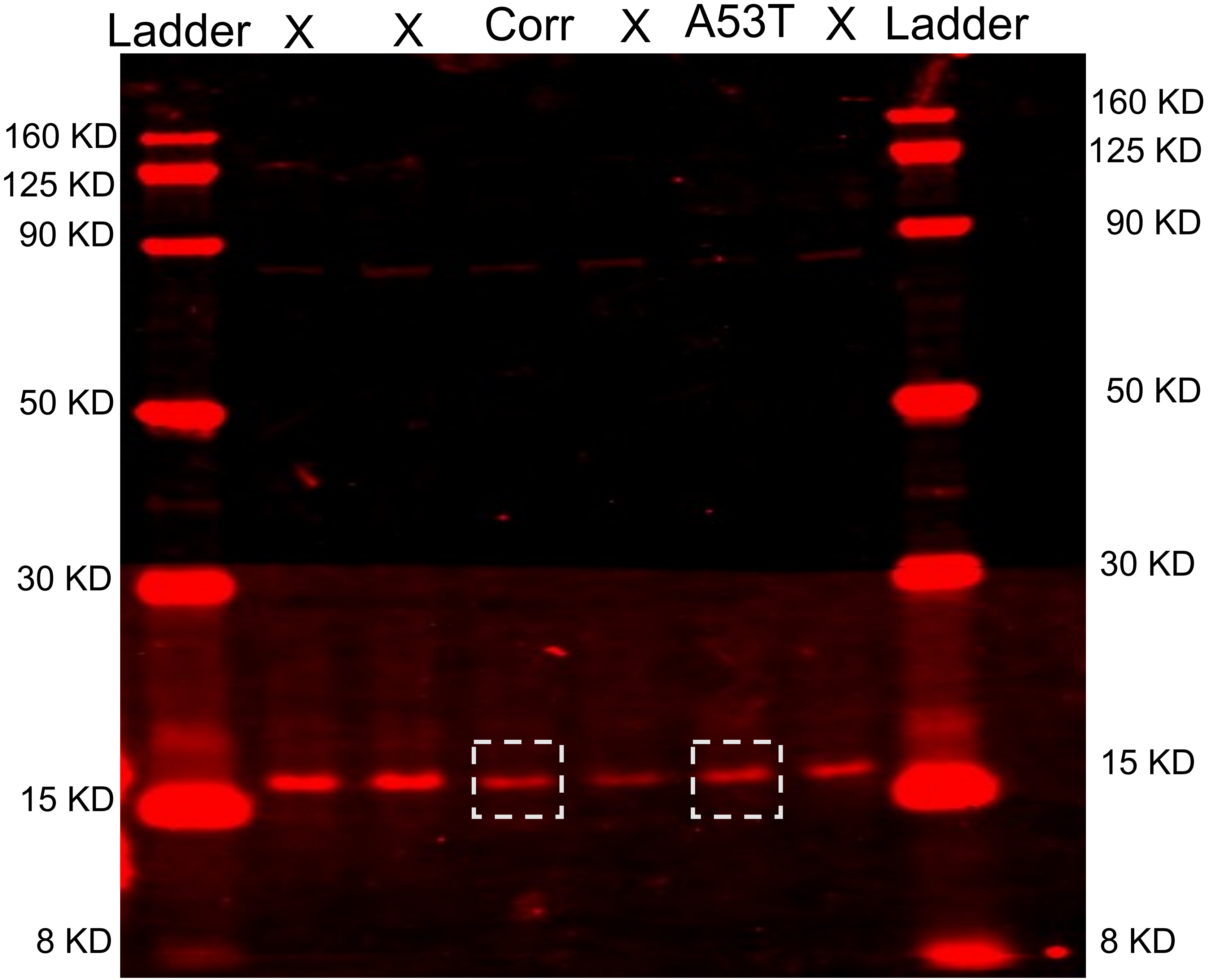

Blot was cut, both pieces included

Figure 4A: Vinculin Panels
A53T – Disease NSC
Corr – Isogenic Control NSC
Imaged with LiCor Odyssey
Dlx System

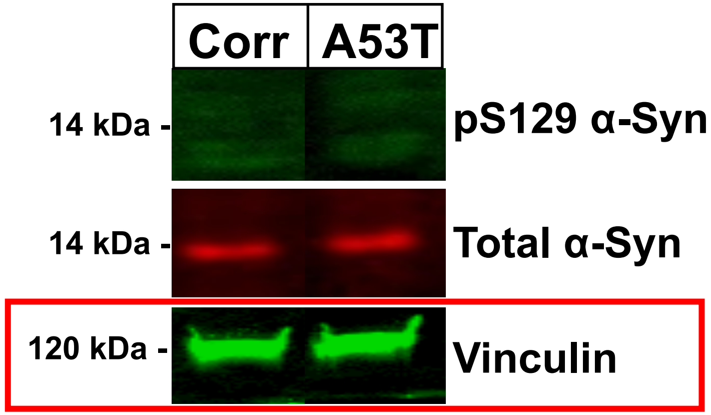

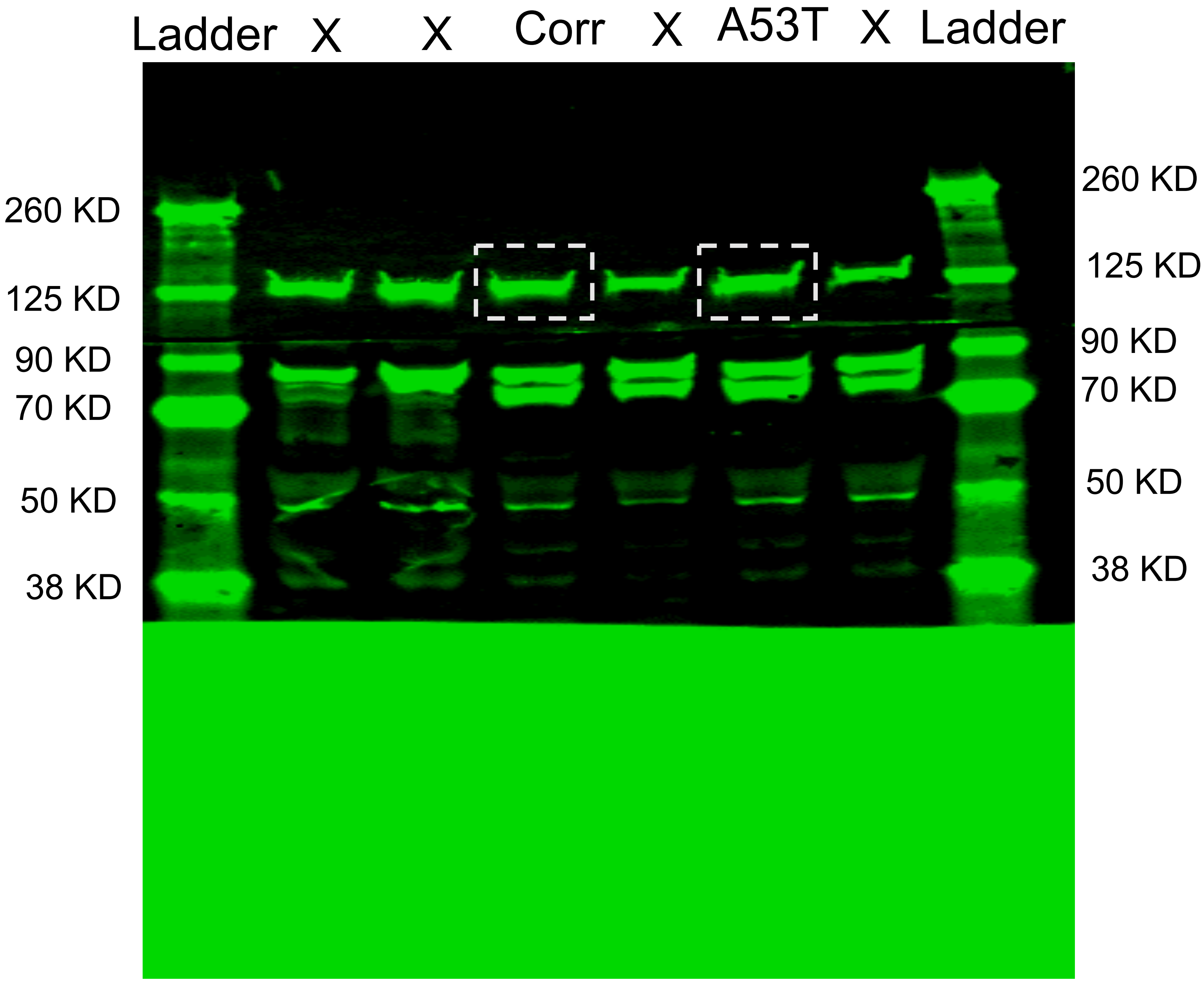

Blot was cut, both pieces included

Figure 4B: pS129 α-Syn Panels
S3 – Disease NSC
KD – Isogenic Control NSC
Imaged with LiCor Odyssey DIx System

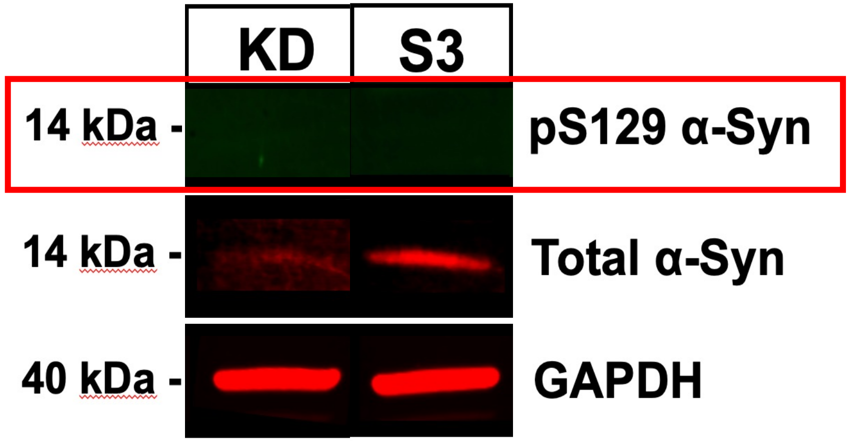

Blot was cut, only bottom piece included

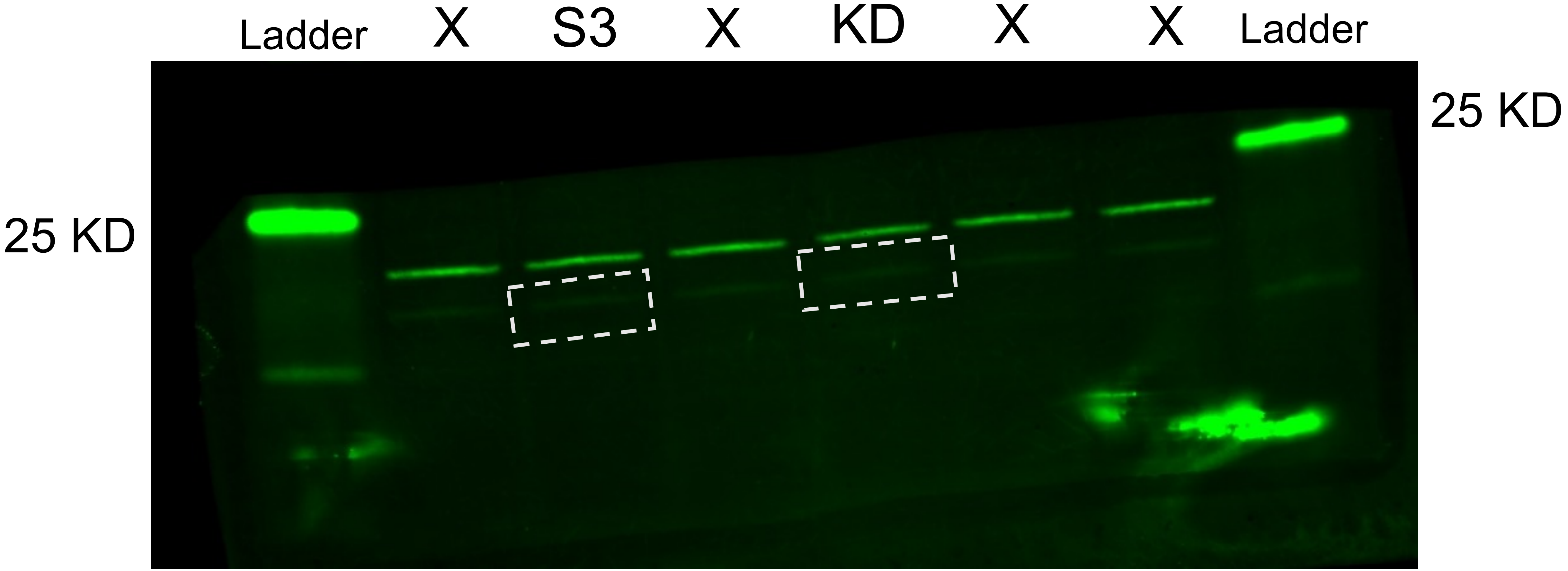

Figure 4B: Total α-Syn Panels
S3 – Disease NSC
KD – Isogenic Control NSC
Imaged with LiCor Odyssey Dlx System

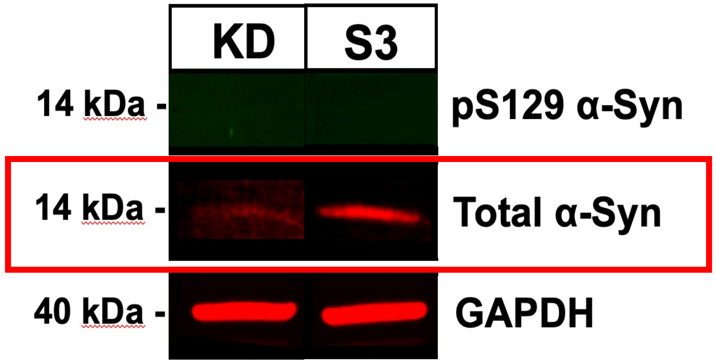

Blot was cut, only bottom piece included

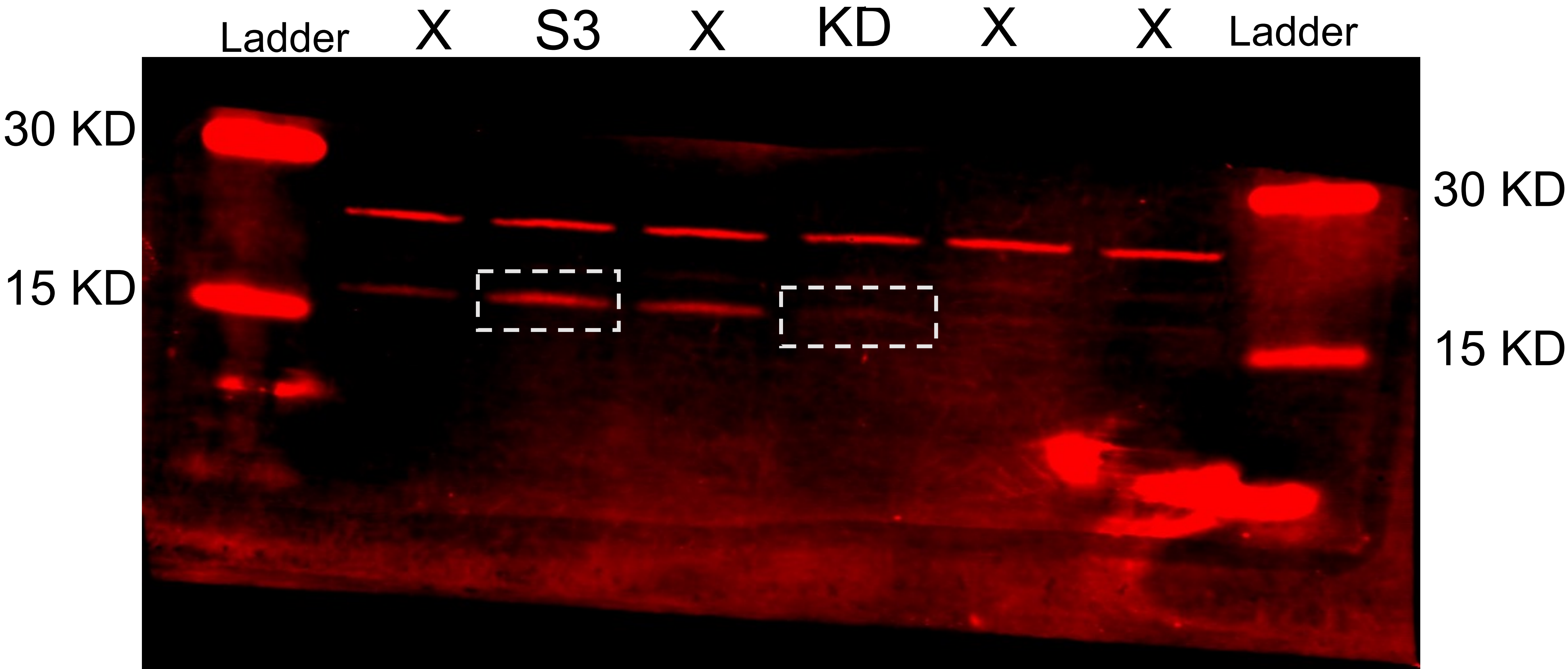

Figure 4B:
GAPDH Panels
S3 – Disease NSC
KD – Isogenic Control NSC
Imaged with LiCor Odyssey Dlx System

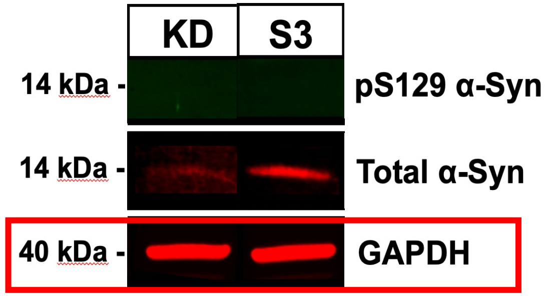

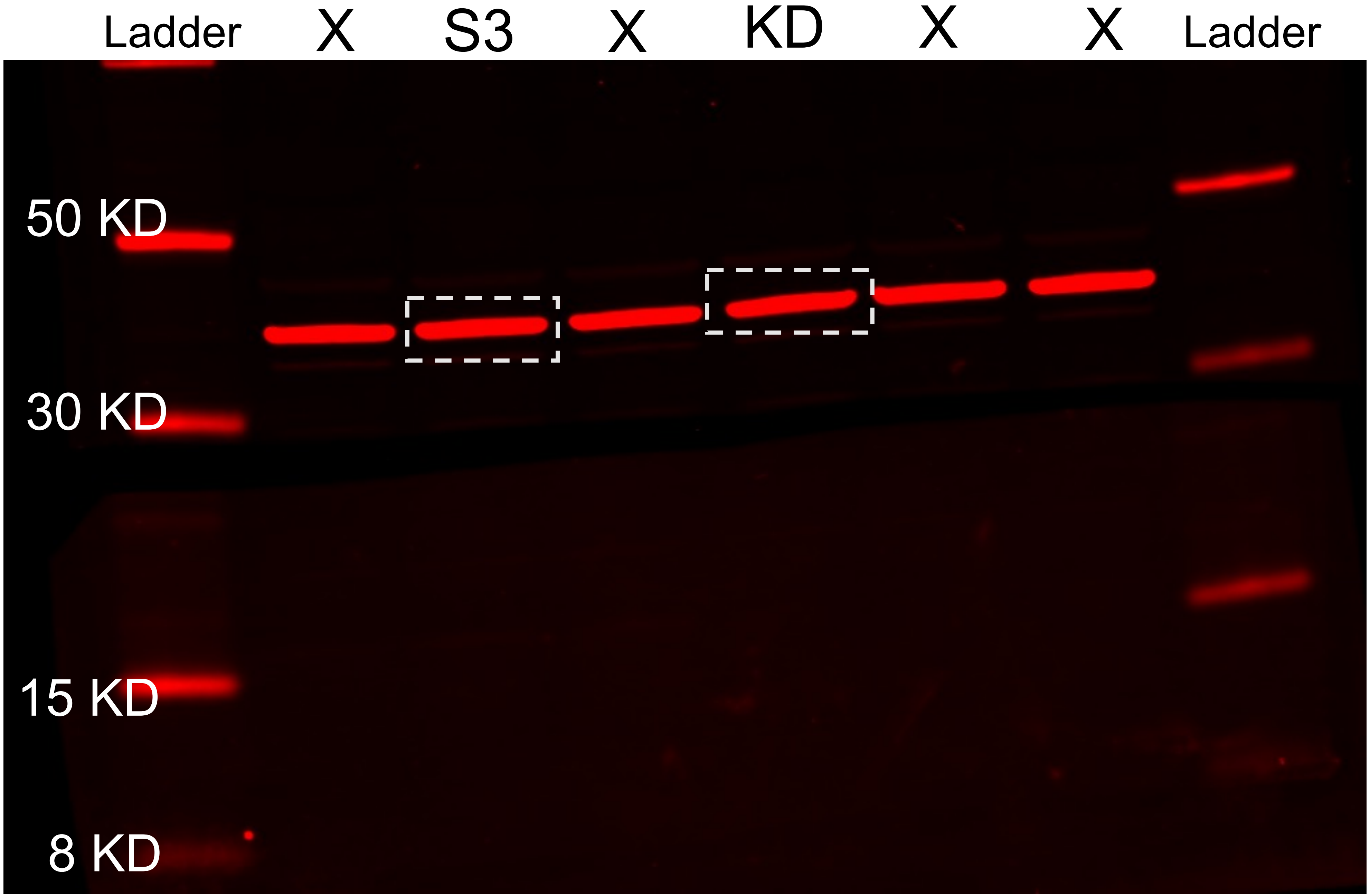

Blot was cut, only bottom piece included

Figure 4E: Vinculin and pS129 α-Syn Panels

A53T – Disease Neurospheres
Corr – Isogenic Control Neurospheres

Imaged with LiCor Odyssey DlX System

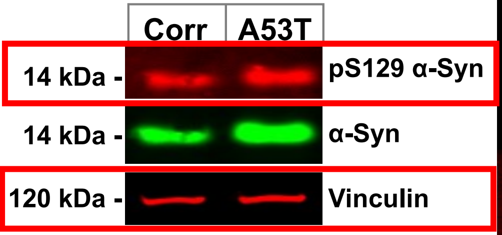

Blot was cut, the two cut pieces are shown

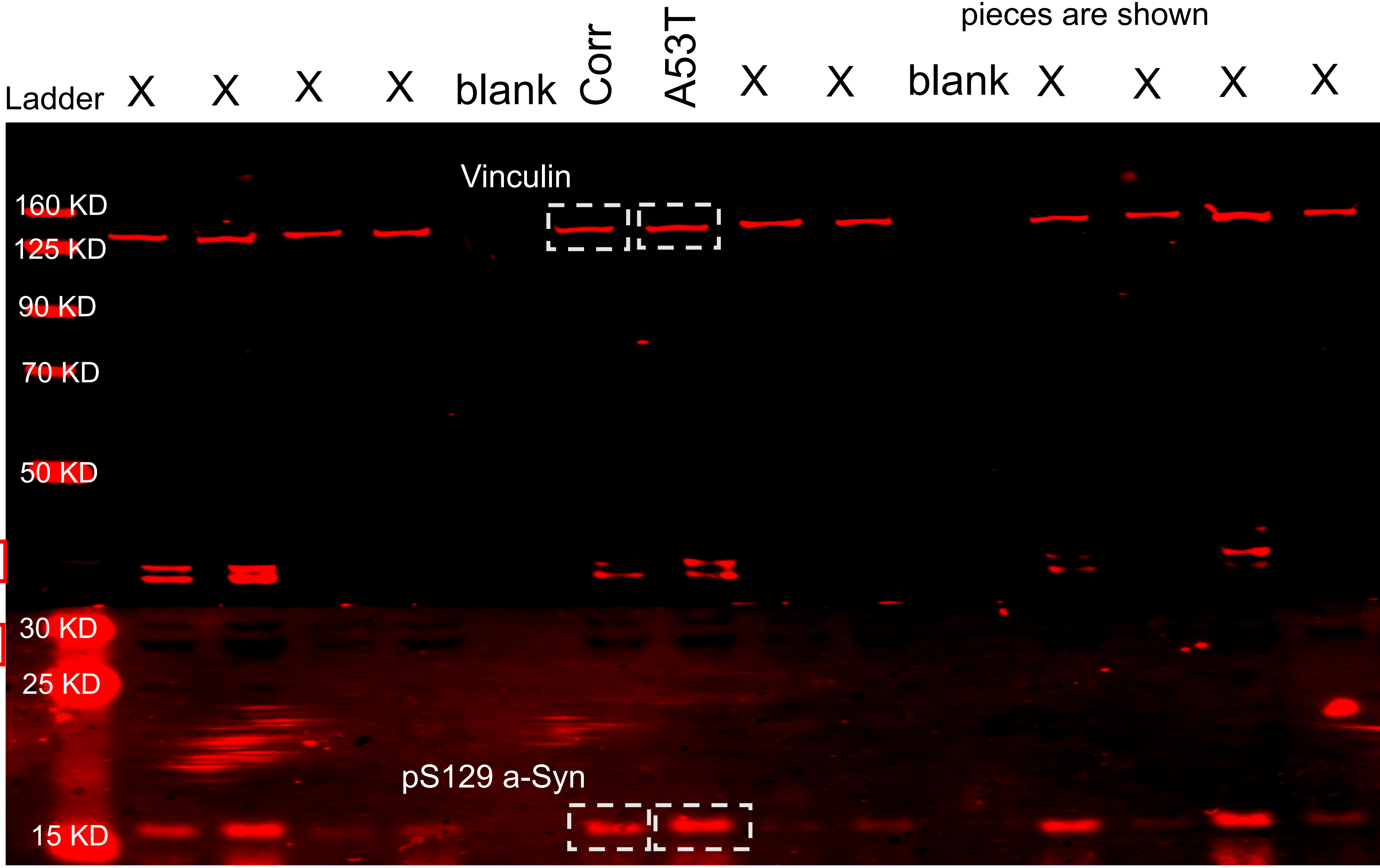

Figure 4E: Total α-Syn Panels

Blot was cut, the two cut pieces are shown

A53T – Disease Neurospheres
Corr – Isogenic Control Neurospheres

Imaged with LiCor Odyssey Dlx System

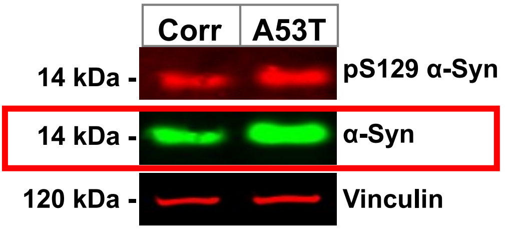

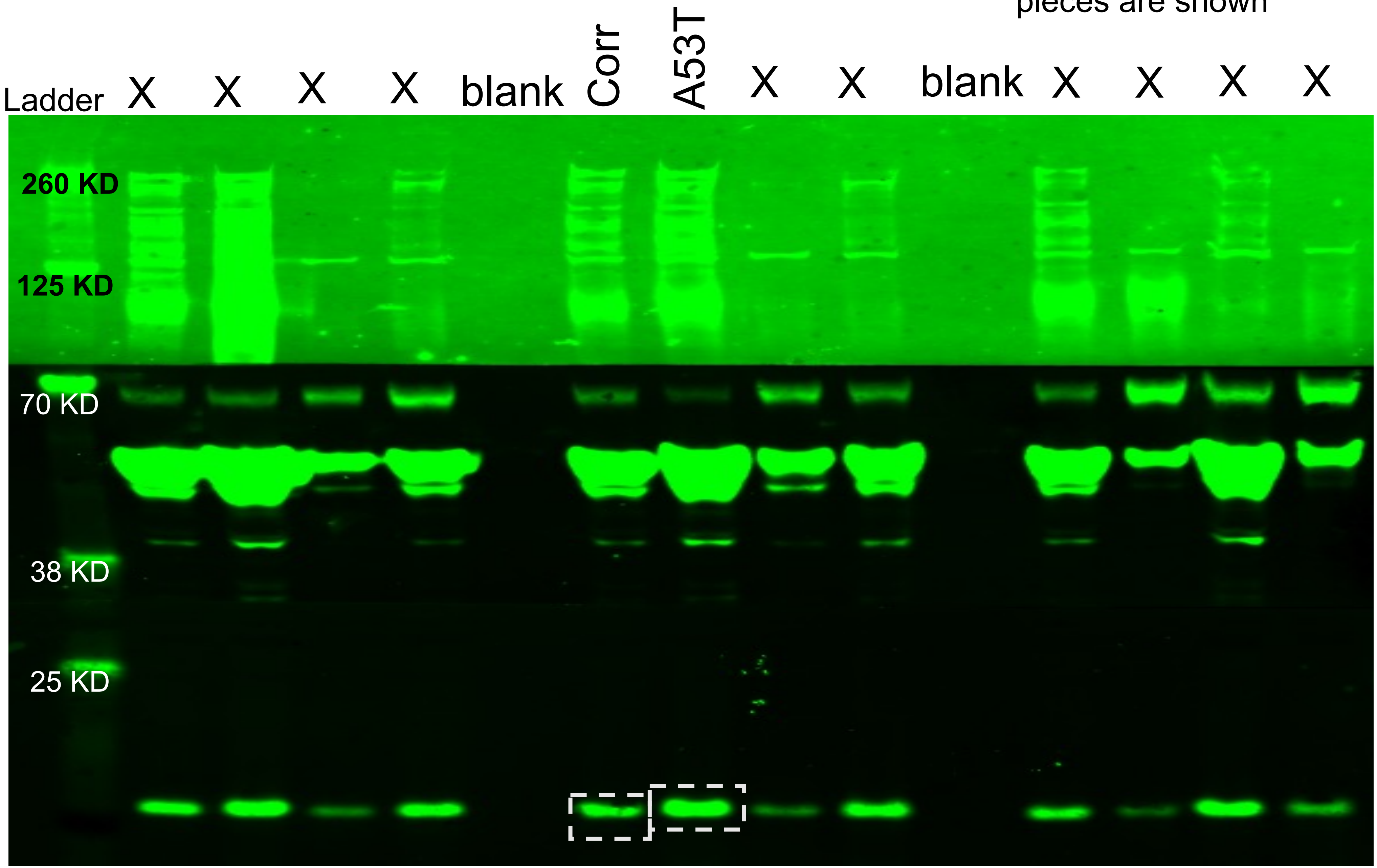

Figure 4F: pS129 α-Syn Panels

S3 – Disease Neurospheres
KD – Isogenic Control Neurospheres

Imaged with LiCor Odyssey Dlx System

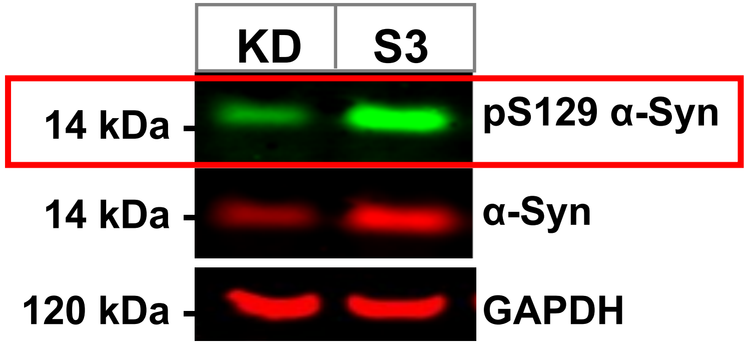

Blot was cut, the two cut pieces are shown

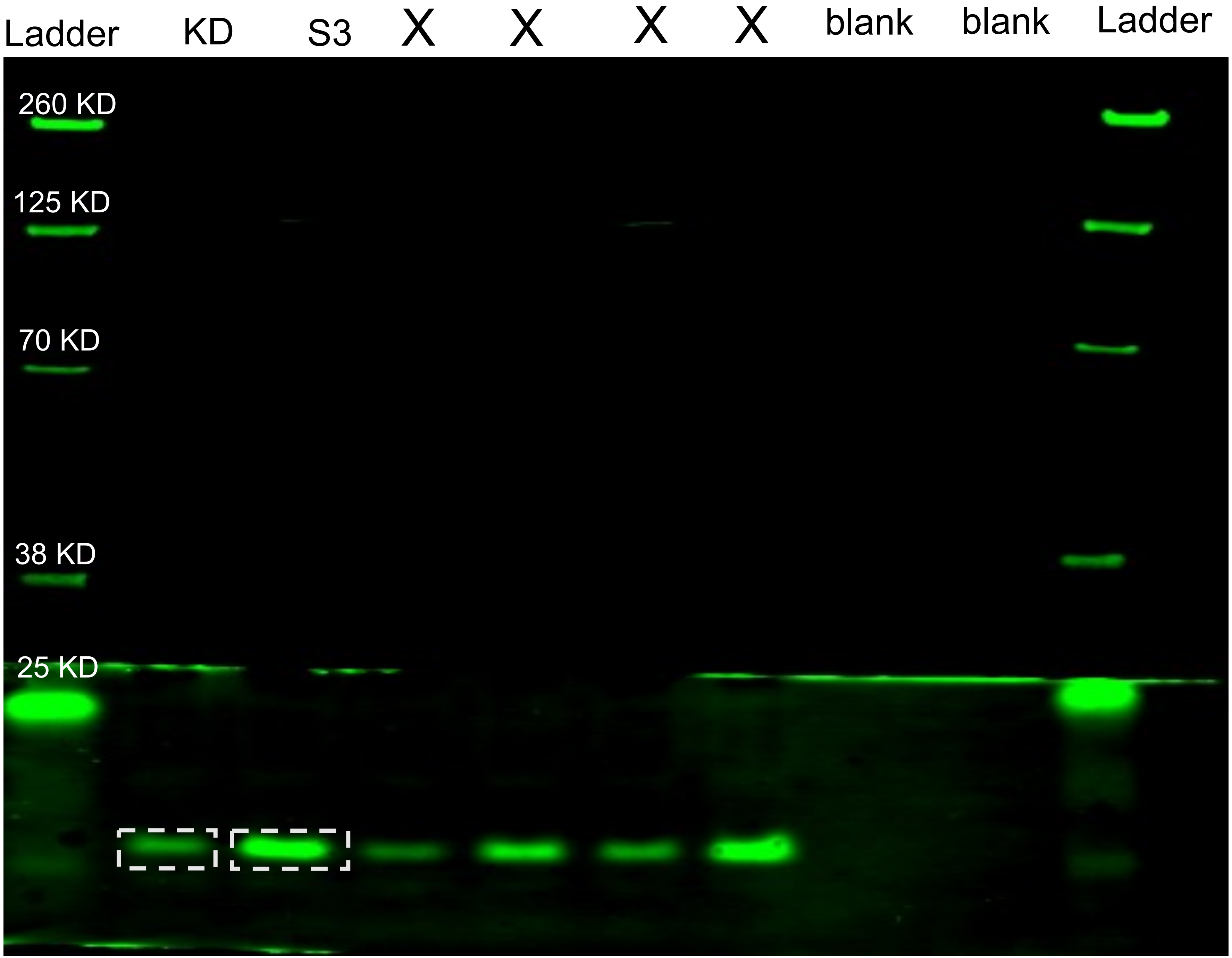

Figure 4F:

Total α-Syn and GAPDH Panels

S3 – Disease Neurospheres

KD – Isogenic Control Neurospheres

Imaged with LiCor Odyssey Dlx System

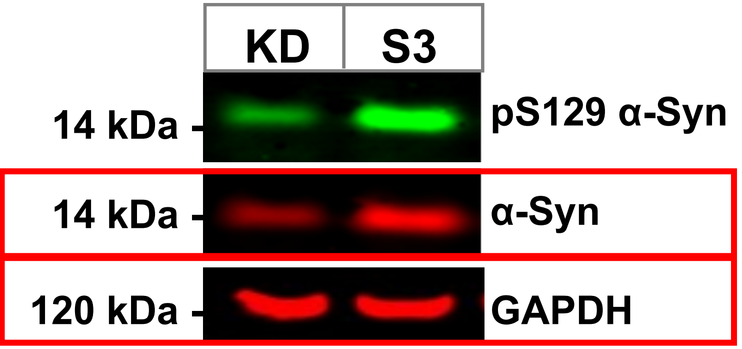

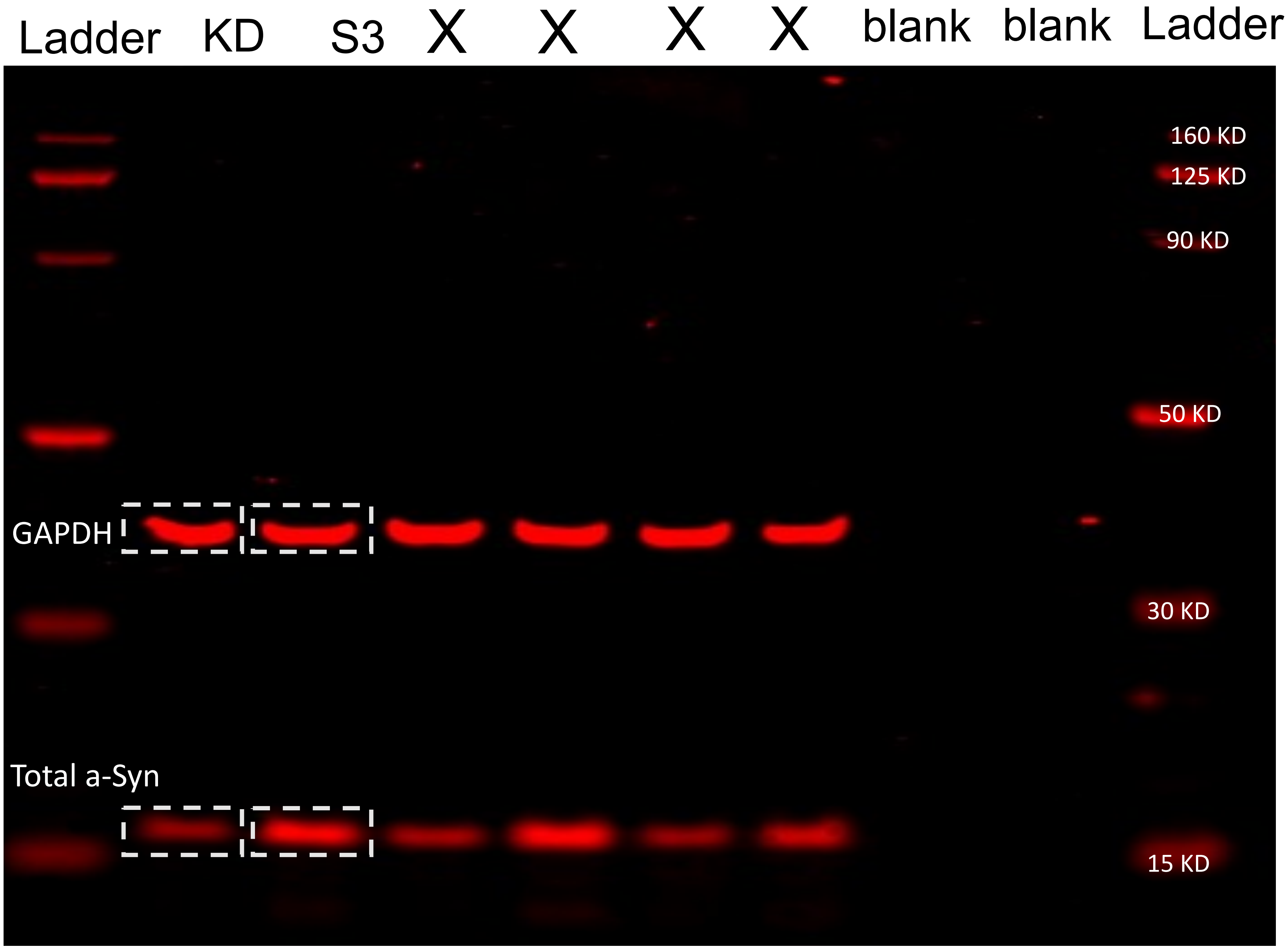

Blot was cut, the two cut pieces are shown

Figure 5C: Vinculin Panels
Imaged with LiCor Odyssey Dlx System

A53T DMSO – Disease Neurospheres treated with DMSO control
A53T Cay10566 – Disease Neurospheres treated with Cay10566
Corr DMSO – Isogenic Control Neurospheres treated with DMSO control
Corr Cay10566 – Isogenic Control Neurospheres treated with Cay10566

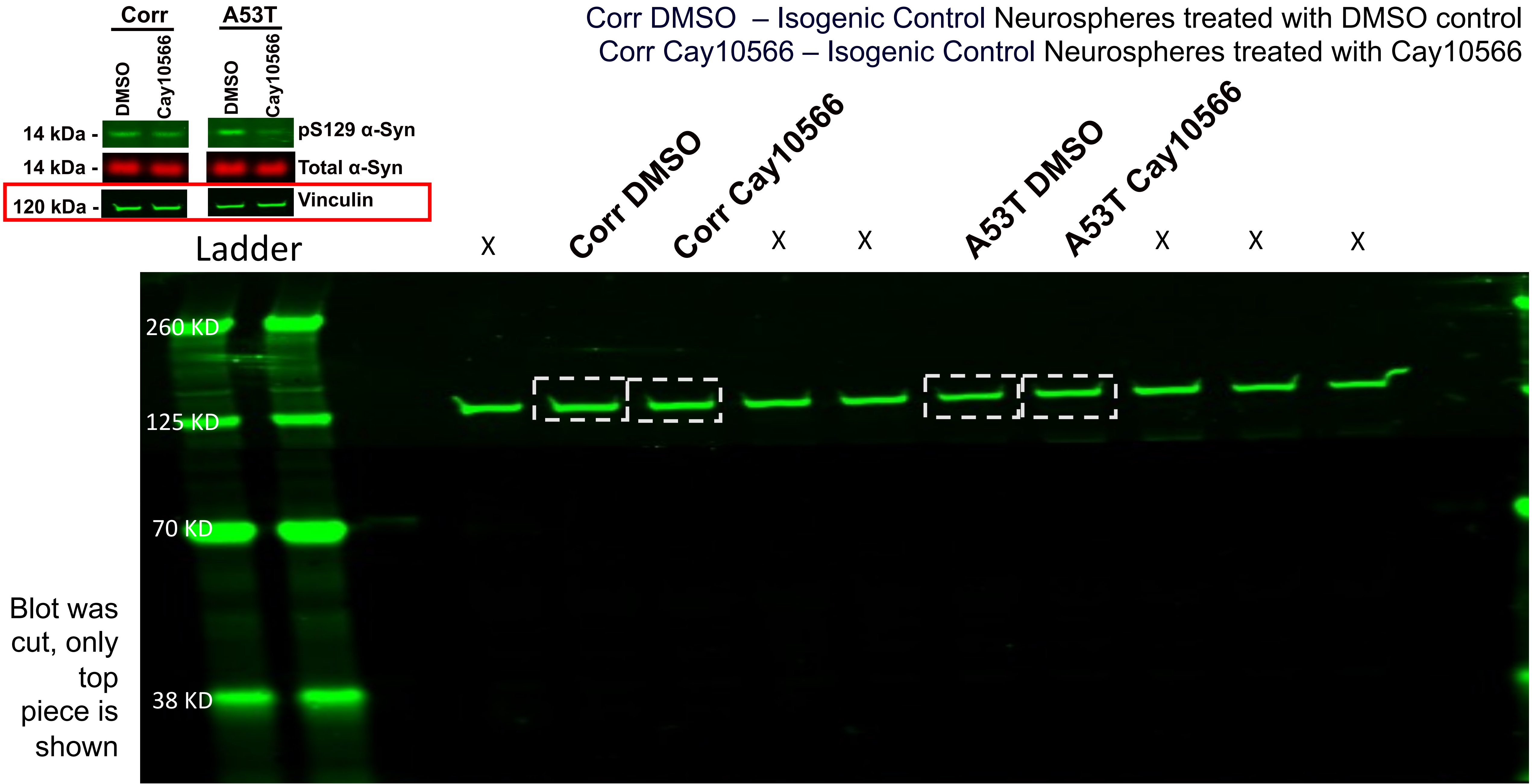

Blot was cut, only top piece is shown

Figure 5C: pS129 a-Syn Panels
Imaged with LiCor Odyssey Dlx System

A53T DMSO – Disease Neurospheres treated with DMSO control
A53T Cay10566 – Disease Neurospheres treated with Cay10566
Corr DMSO – Isogenic Control Neurospheres treated with DMSO control
Corr Cay10566 – Isogenic Control Neurospheres treated with Cay10566

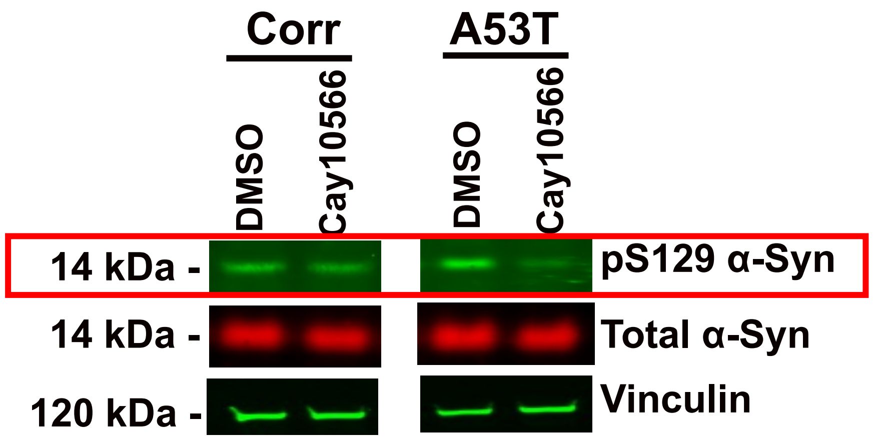

Blot was cut, only bottom piece is shown

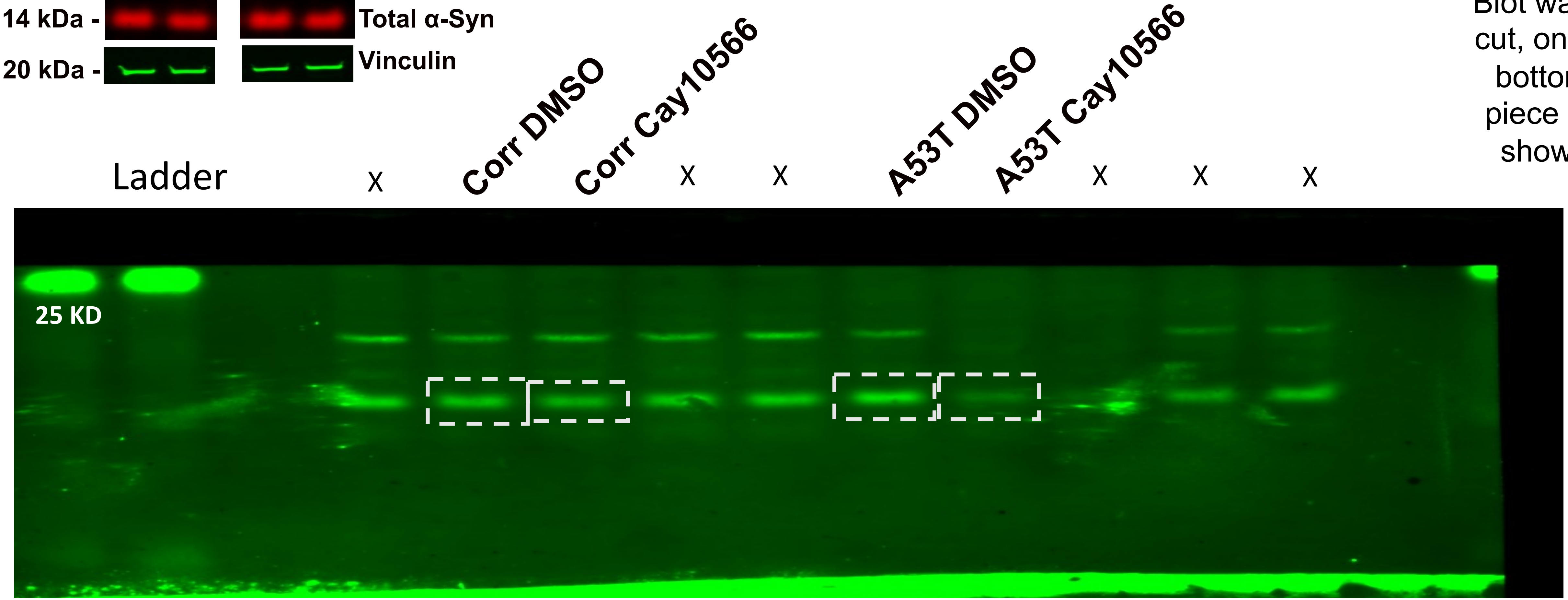

Figure 5C: Total a-Syn Panels
Imaged with LiCor Odyssey Dlx System

A53T DMSO – Disease Neurospheres treated with DMSO control
A53T Cay10566 – Disease Neurospheres treated with Cay10566
Corr DMSO – Isogenic Control Neurospheres treated with DMSO control
Corr Cay10566 – Isogenic Control Neurospheres treated with Cay10566

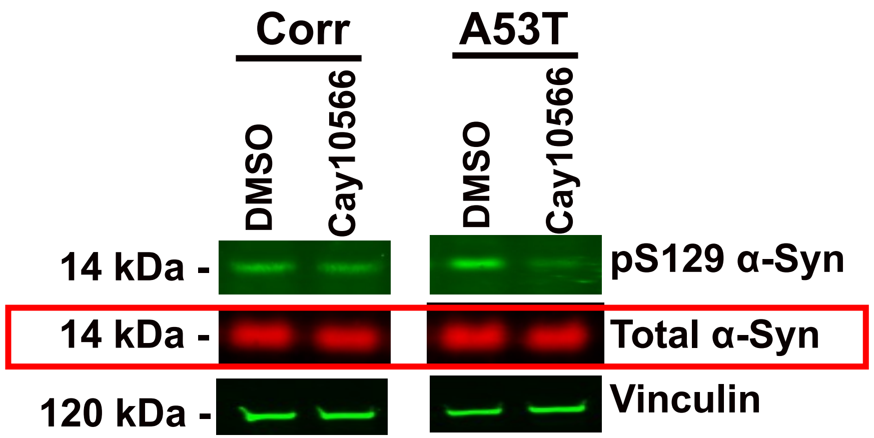

Blot was cut, only bottom piece is shown

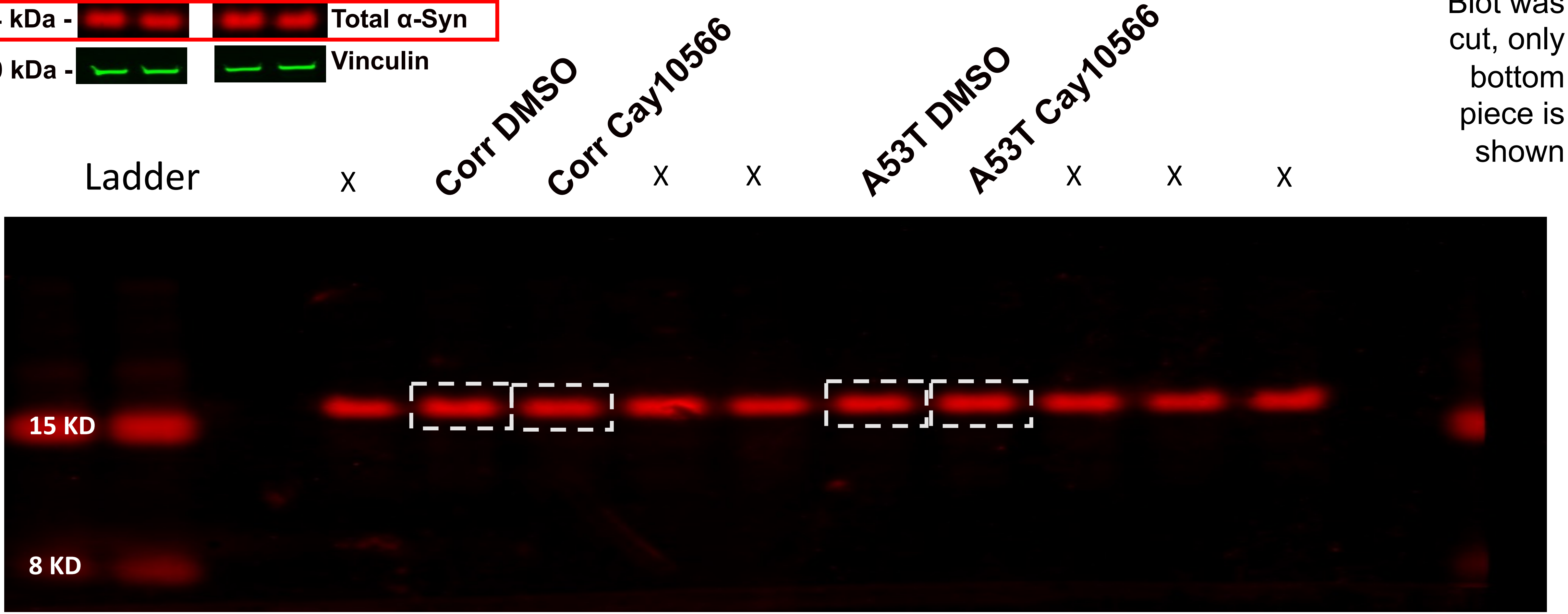

Figure 5D: GAPDH Panels
Imaged with LiCor Odyssey Dlx System

S3 DMSO – Disease Neurospheres treated with DMSO control
S3 Cay10566 – Disease Neurospheres treated with Cay10566
KD DMSO – Isogenic Control Neurospheres treated with DMSO control
KD Cay10566 – Isogenic Control Neurospheres treated with Cay10566

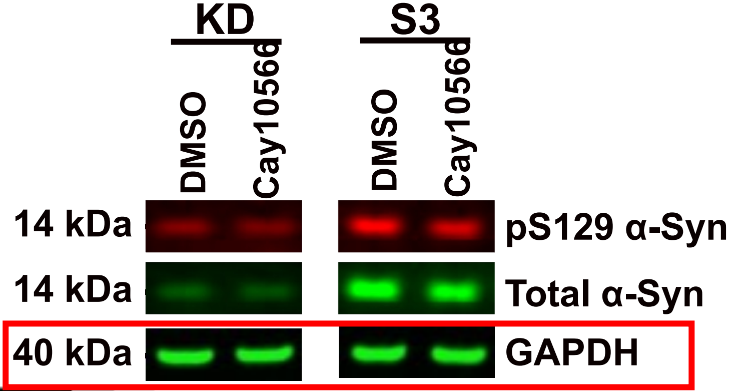

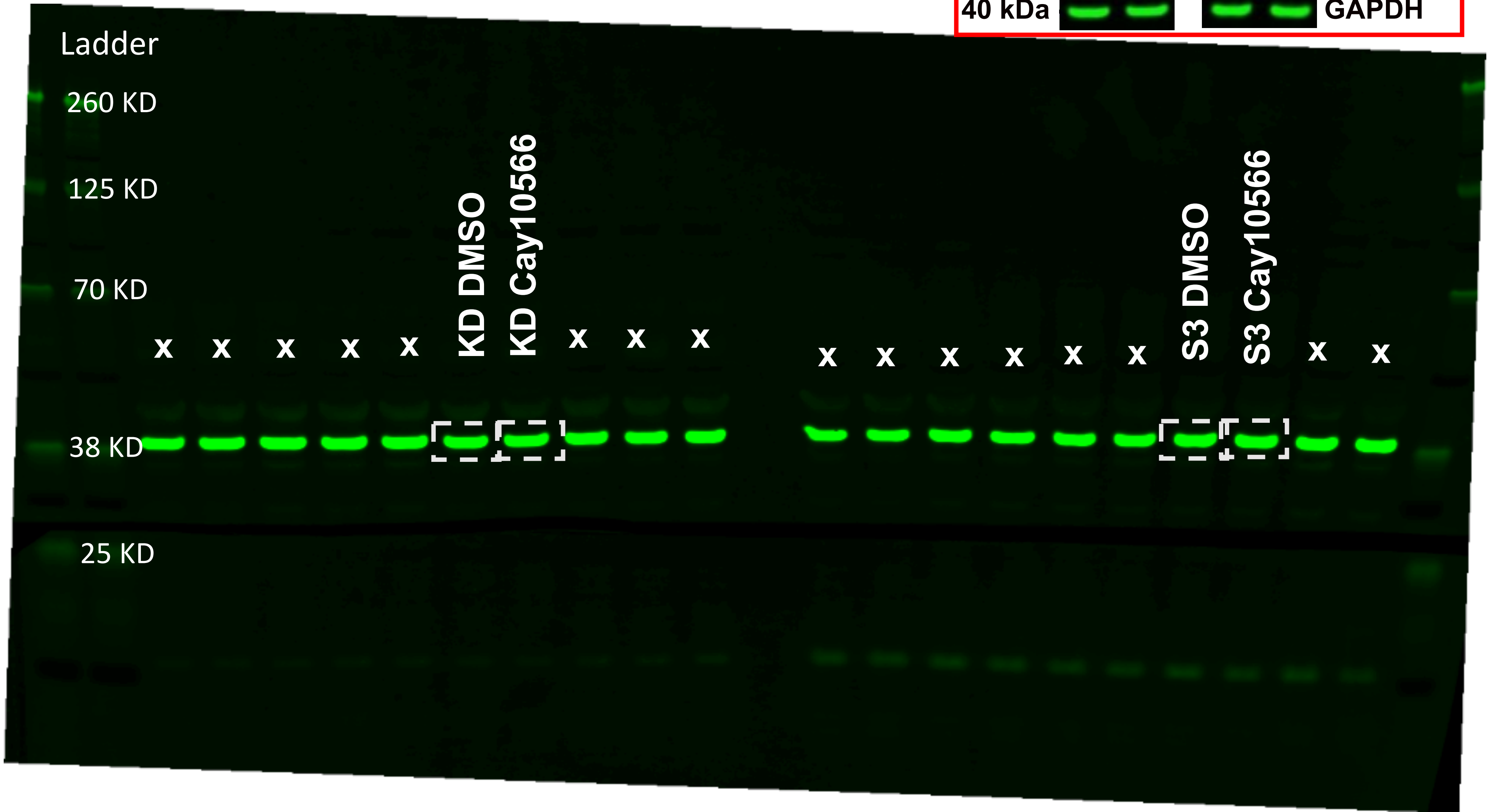

Blot was cut, only top piece is shown

Figure 5D: pS129 a-Syn Panels

Imaged with LiCor Odyssey Dlx System

S3 DMSO – Disease Neurospheres treated with DMSO control

S3 Cay10566 – Disease Neurospheres treated with Cay10566

KD DMSO – Isogenic Control Neurospheres treated with DMSO control

KD Cay10566 – Isogenic Control Neurospheres treated with Cay10566

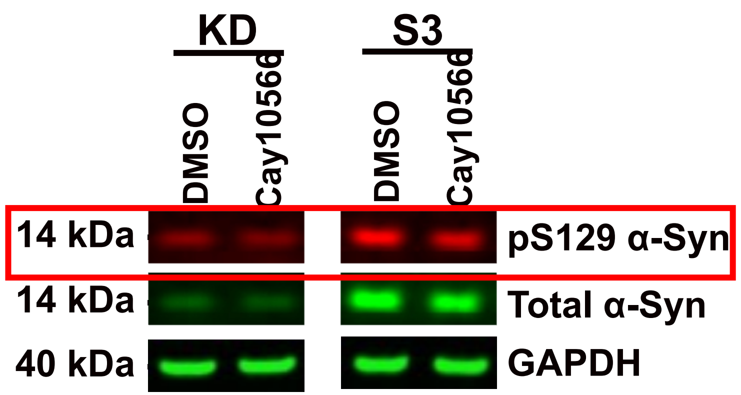

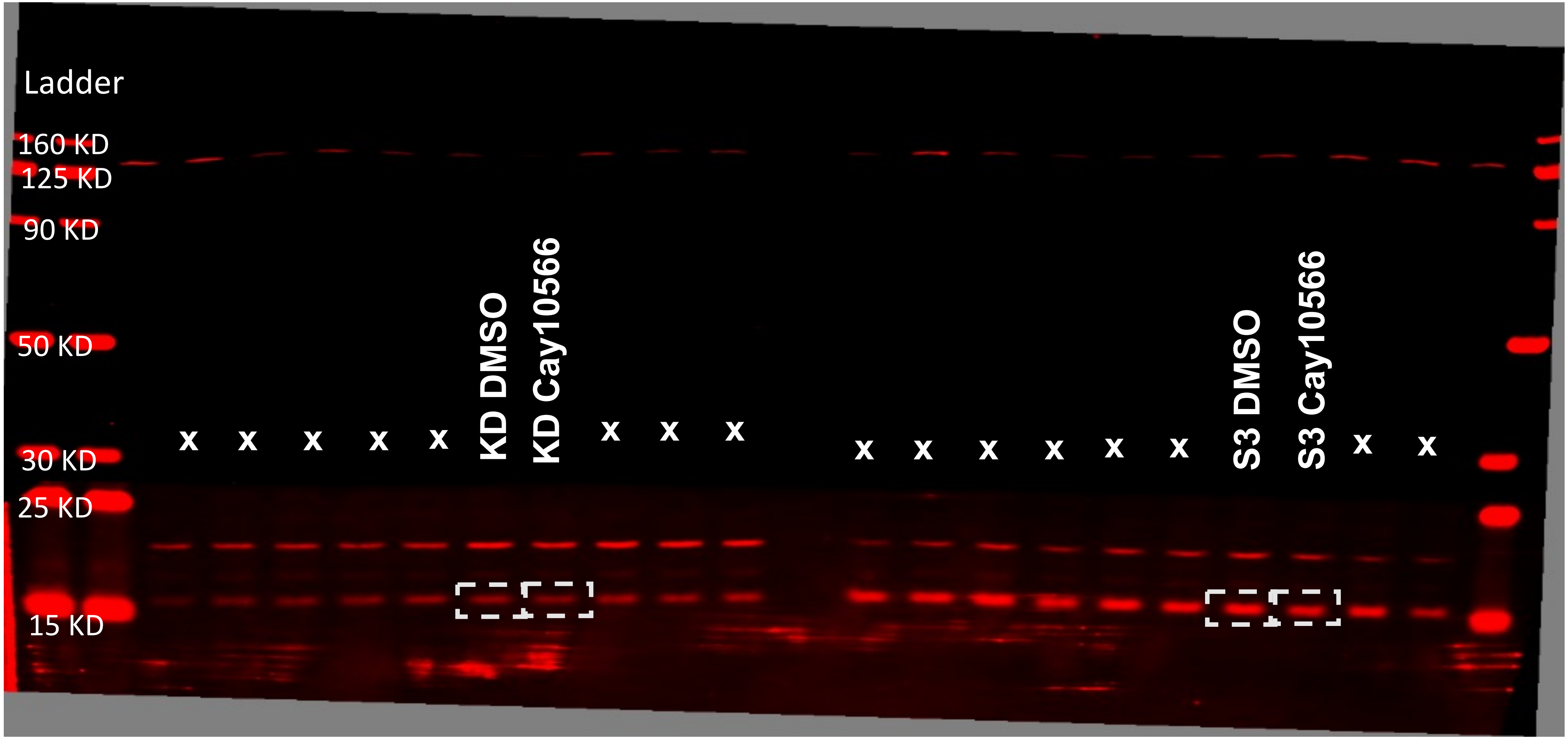

Blot was cut, both pieces shown

Figure 5D: Total a-Syn Panels

Imaged with LiCor Odyssey Dlx System

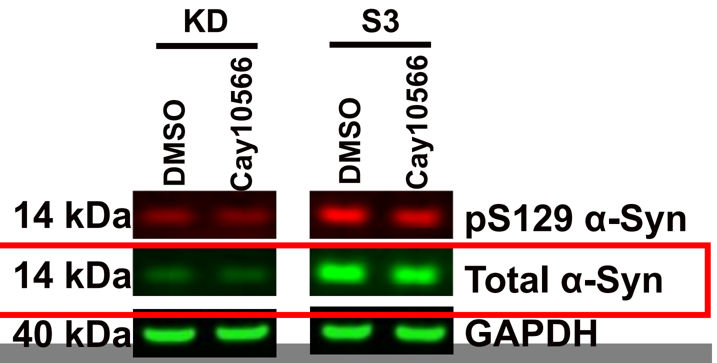

S3 DMSO – Disease Neurospheres treated with DMSO control

S3 Cay10566 – Disease Neurospheres treated with Cay10566

KD DMSO – Isogenic Control Neurospheres treated with DMSO control

KD Cay10566 – Isogenic Control Neurospheres treated with Cay10566

Blot was cut, both pieces shown

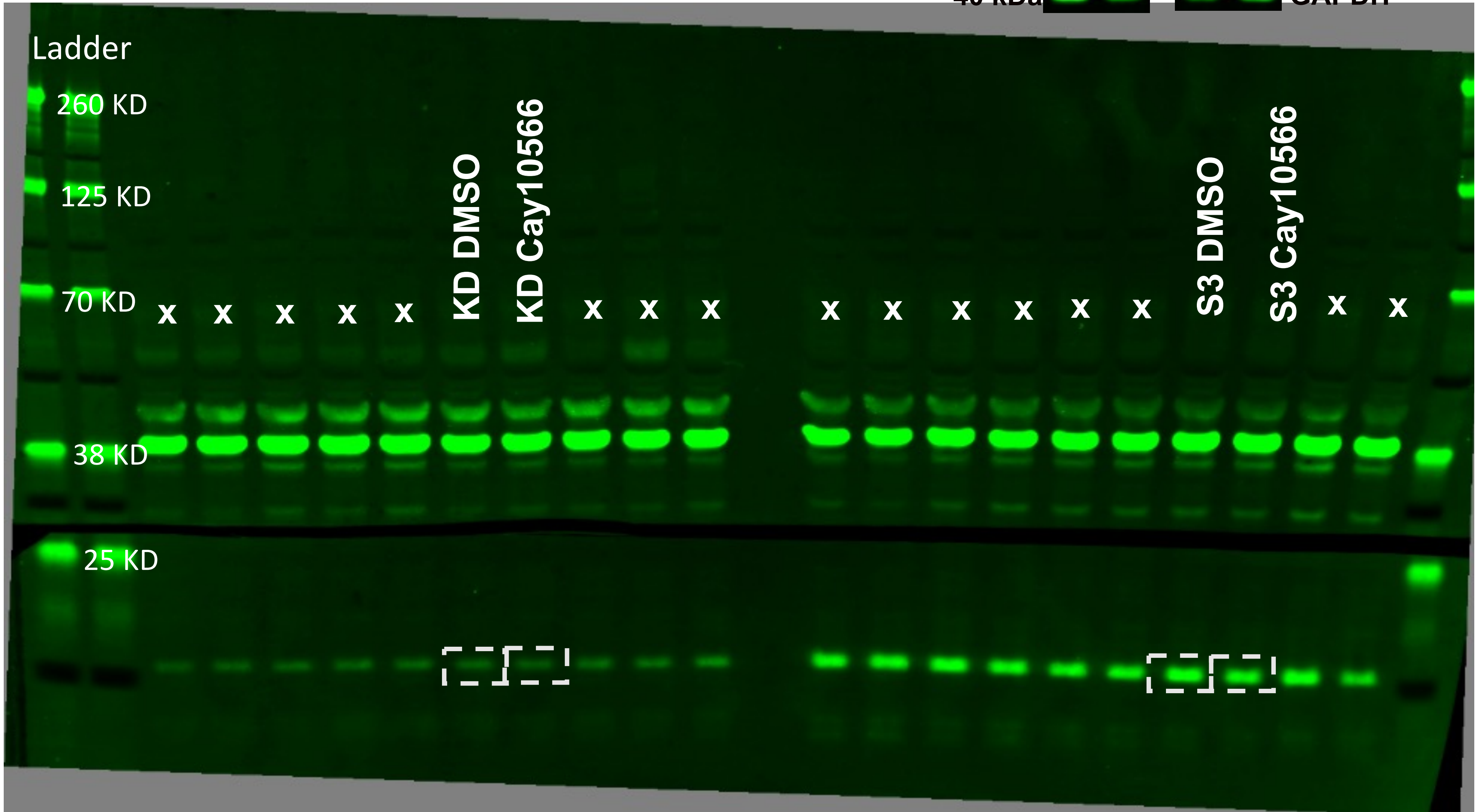

Supplementary Figure 8: Synapsin-I Panels
Imaged with LiCor Odyssey DIx System

S3 DMSO – Disease Neurospheres treated with DMSO control
S3 Cay10566 – Disease Neurospheres treated with Cay10566
KD DMSO – Isogenic Control Neurospheres treated with DMSO control
KD Cay10566 – Isogenic Control Neurospheres treated with Cay10566

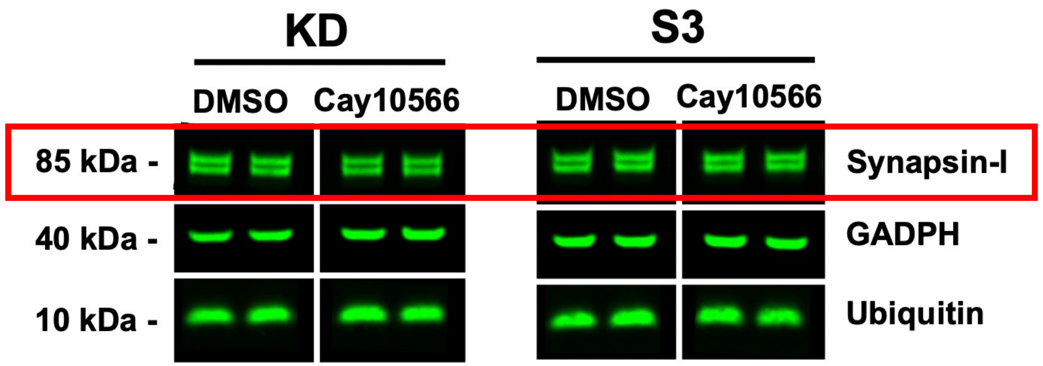

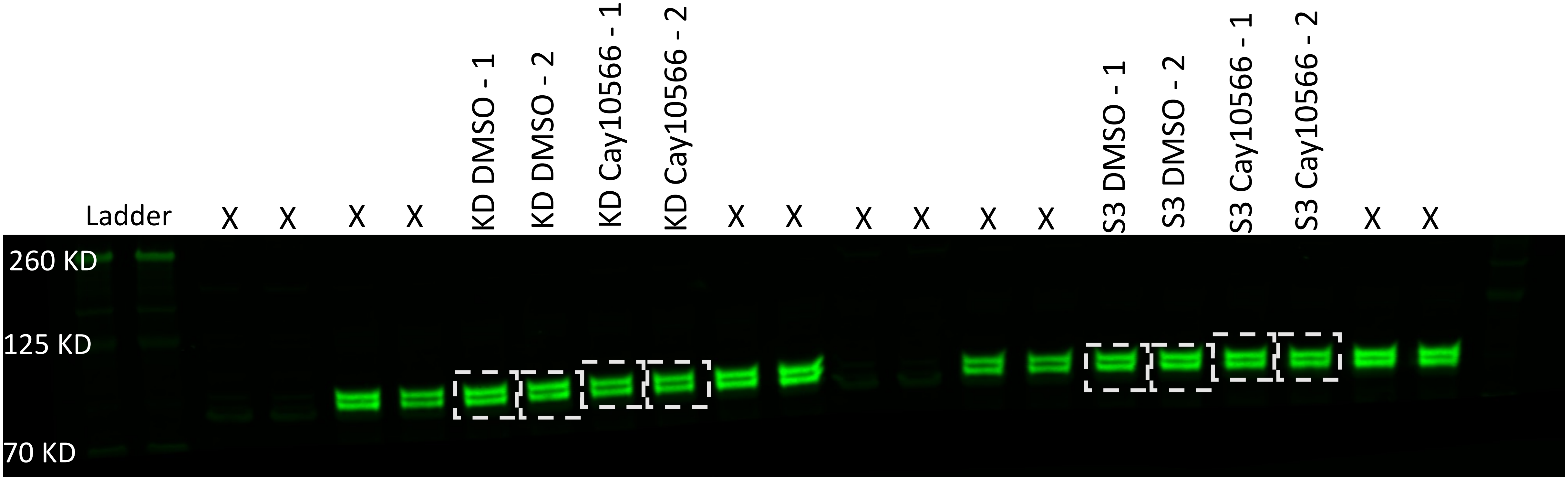

Blot was cut, only top piece shown

Supplementary Figure 8: GAPDH Panels

Imaged with LiCor Odyssey DIx System

S3 DMSO – Disease Neurospheres treated with DMSO control

S3 Cay10566 – Disease Neurospheres treated with Cay10566

KD DMSO – Isogenic Control Neurospheres treated with DMSO control

KD Cay10566 – Isogenic Control Neurospheres treated with Cay10566

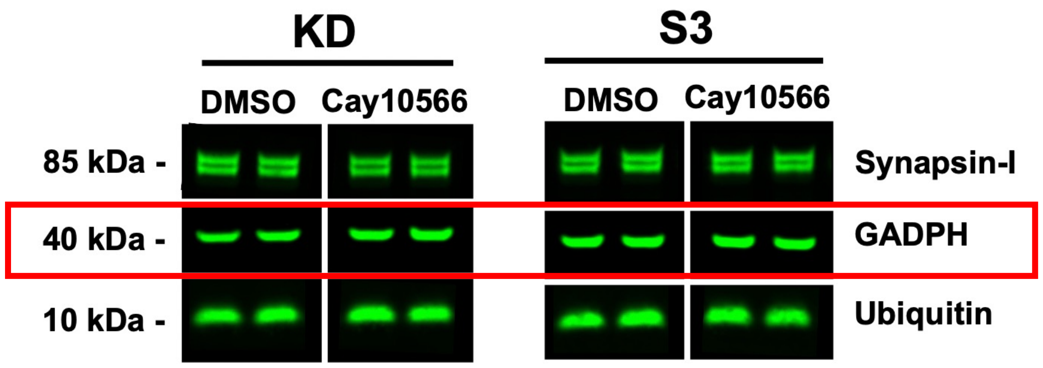

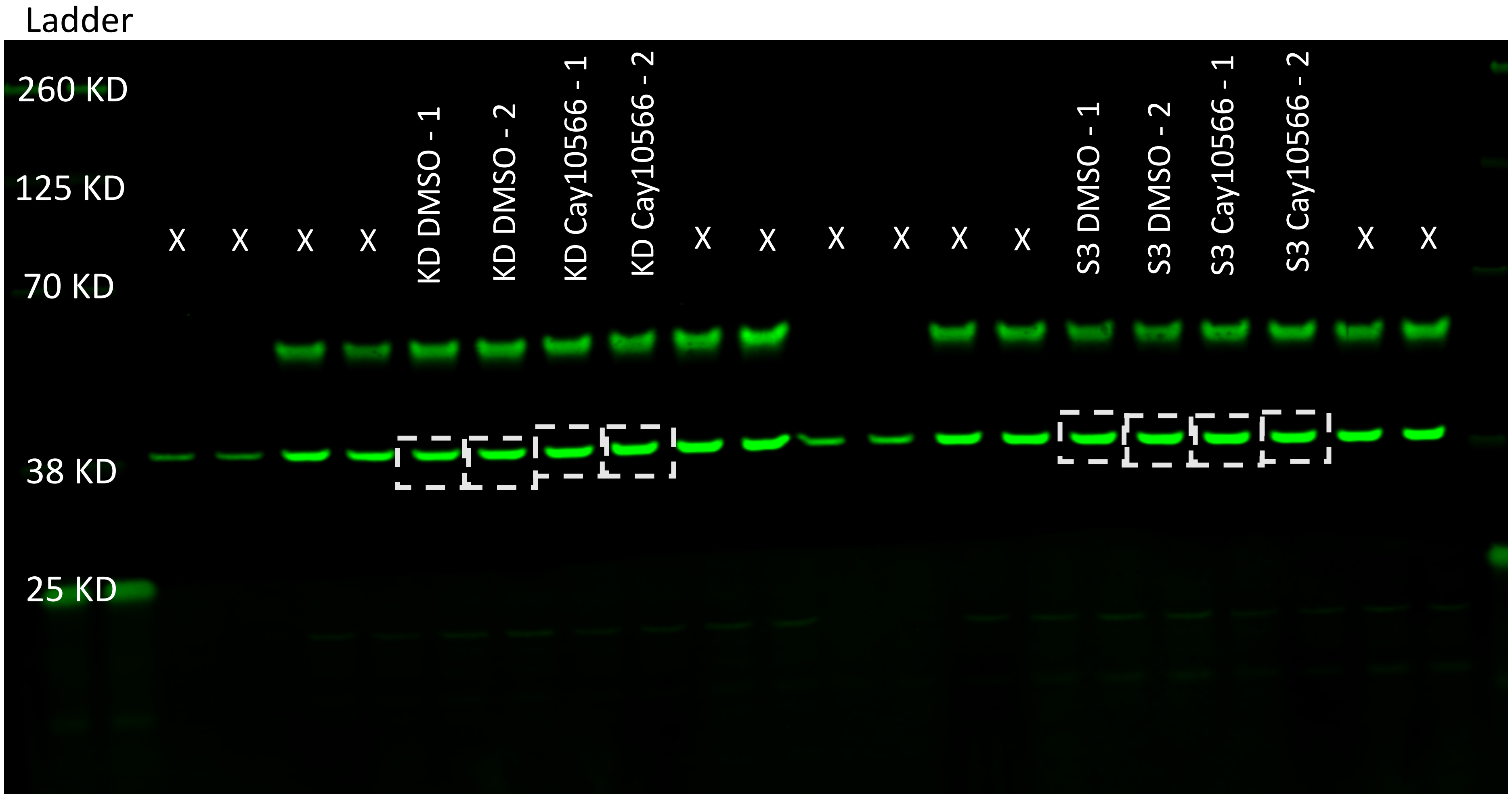

Blot was cut, both pieces shown

Supplementary Figure 8: Ubiquitin Panels
Imaged with LiCor Odyssey Dlx System

S3 DMSO – Disease Neurospheres treated with DMSO control
S3 Cay10566 – Disease Neurospheres treated with Cay10566
KD DMSO – Isogenic Control Neurospheres treated with DMSO control
KD Cay10566 – Isogenic Control Neurospheres treated with Cay10566

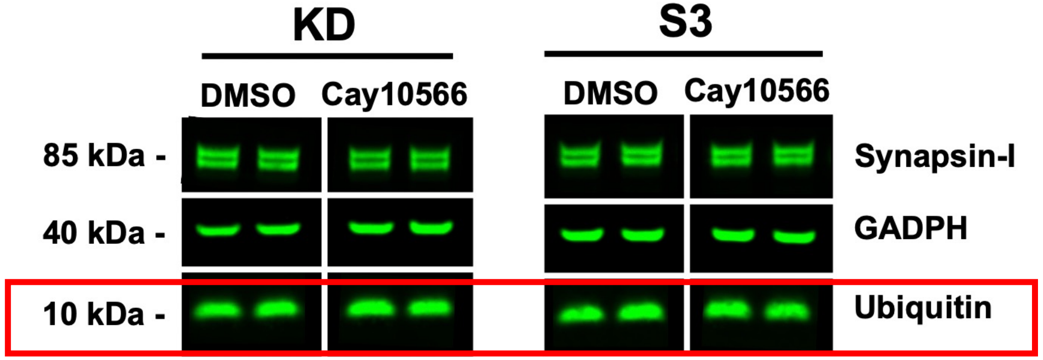

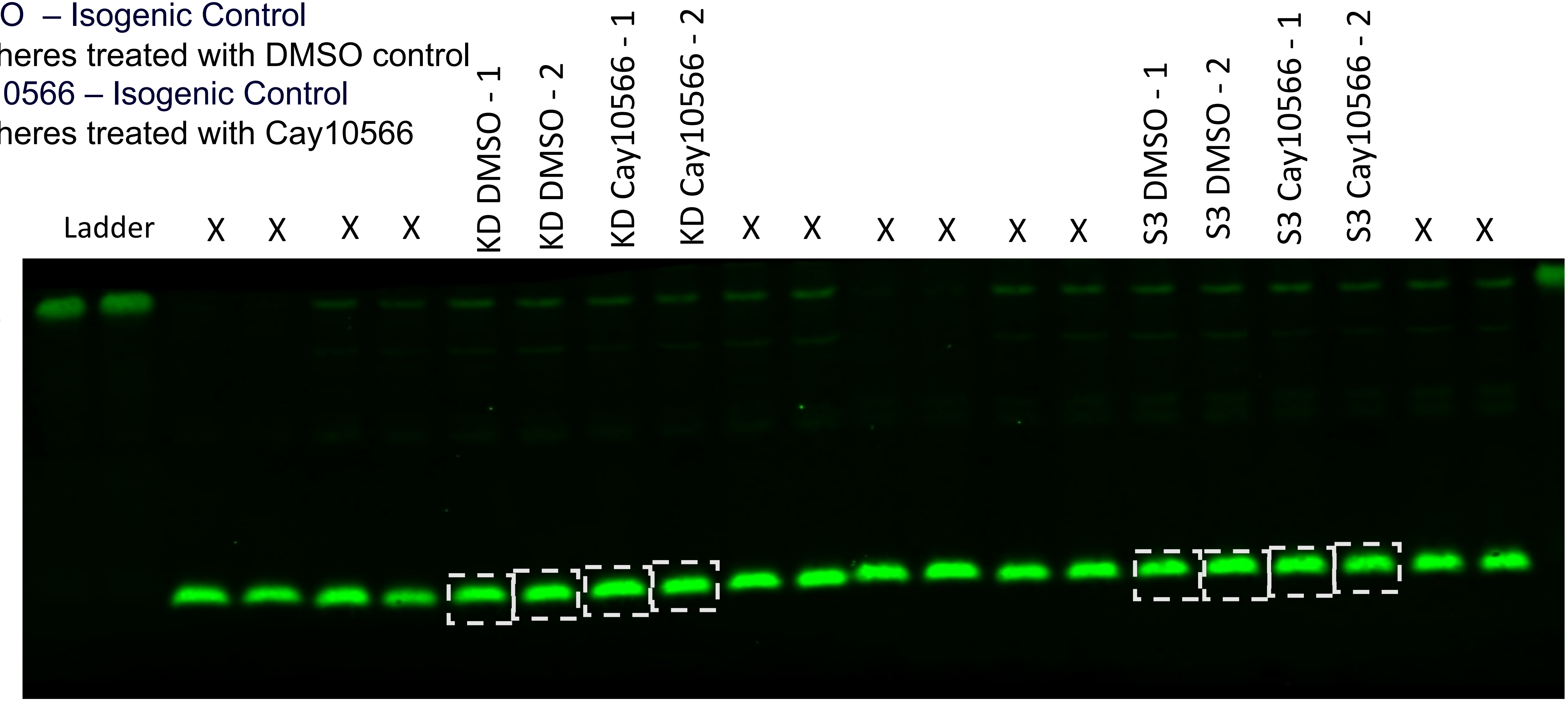

Blot was cut, only bottom piece shown
